# TADF: Enabling luminescent copper(i) coordination compounds for light-emitting electrochemical cells

**DOI:** 10.1039/d1tc04028f

**Published:** 2021-10-12

**Authors:** Catherine E. Housecroft, Edwin C. Constable

**Affiliations:** Department of Chemistry, University of Basel Mattenstrasse 24a, BPR 1096 4058-Basel Switzerland catherine.housecroft@unibas.ch

## Abstract

The last decade has seen a surge of interest in the emissive behaviour of copper(i) coordination compounds, both neutral compounds that may have applications in organic light-emitting doides (OLEDs) and copper-based ionic transition metal complexes (Cu-iTMCs) with potential use in light-emitting electrochemical cells (LECs). One of the most exciting features of copper(i) coordination compounds is their possibility to exhibit thermally activated delayed fluorescence (TADF) in which the energy separation of the excited singlet (S_1_) and excited triplet (T_1_) states is very small, permitting intersystem crossing (ISC) and reverse intersystem crossing (RISC) to occur at room temperature without the requirement for the large spin–orbit coupling inferred by the presence of a heavy metal such as iridium. In this review, we focus mainly in Cu-iTMCs, and illustrate how the field of luminescent compounds and those exhibiting TADF has developed. Copper(i) coordination compounds that class as Cu-iTMCs include those containing four-coordinate [Cu(P^P)(N^N)]^+^ (P^P = large-bite angle bisphosphane, and N^N is typically a diimine), [Cu(P)_2_(N^N)]^+^ (P = monodentate phosphane ligand), [Cu(P)(tripodal-N_3_)]^+^, [Cu(P)(N^N)(N)]^+^ (N = monodentate N-donor ligand), [Cu(P^P)(N^S)]^+^ (N^S = chelating N,S-donor ligand), [Cu(P^P)(P^S)]^+^ (P^S = chelating P,S-donor ligand), [Cu(P^P)(NHC)]^+^ (NHC = N-heterocyclic carbene) coordination domains, dinuclear complexes with P^P and N^N ligands, three-coordinate [Cu(N^N)(NHC)]^+^ and two-coordinate [Cu(N)(NHC)]^+^ complexes. We pay particular attention to solid-state structural features, *e.g.* π-stacking interactions and other inter-ligand interactions, which may impact on photoluminescence quantum yields. Where emissive Cu-iTMCs have been tested in LECs, we detail the device architectures, and this emphasizes differences which make it difficult to compare LEC performances from different investigations.

## Abstract 2

In this review, we illustrate how the field of luminescent copper(i) compounds has developed with a focus on ionic copper(i) complexes and those exhibiting thermally-activated delayed fluorescence (TADF).

## Graphical abstract

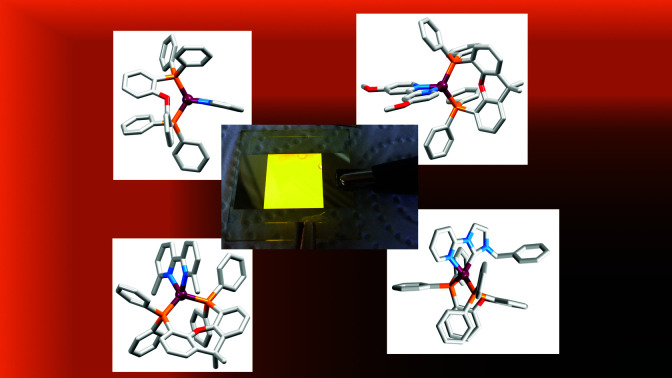

## Introduction

### Lighting: the 21st century CE landscape

In 2015, the United Nations Member States adopted the 2030 Agenda for Sustainable Development with 17 sustainable development goals (SDGs) identified.^1^ The SDGs address all aspects of life on our planet and SDG 7 is concerned with the generation of clean and sustainable energy and the establishment of efficient technologies for energy consumption. A major user of the world's energy is lighting, which accounts for 15% of the energy consumption and 5% of the produced greenhouse gases.^2^ Long-established lighting technologies such as the incandescent lamp are extremely inefficient and more efficient devices such as fluorescent tubes have unsustainable and ecologically harmful materials demands. Within Europe, efficient solid-state lighting devices such as light-emitting diodes (LEDs) and organic light-emitting diodes (OLEDs) have essentially replaced the earlier technologies.^3^

### The light-emitting electrochemical cell (LEC)

**Fig. 1:**
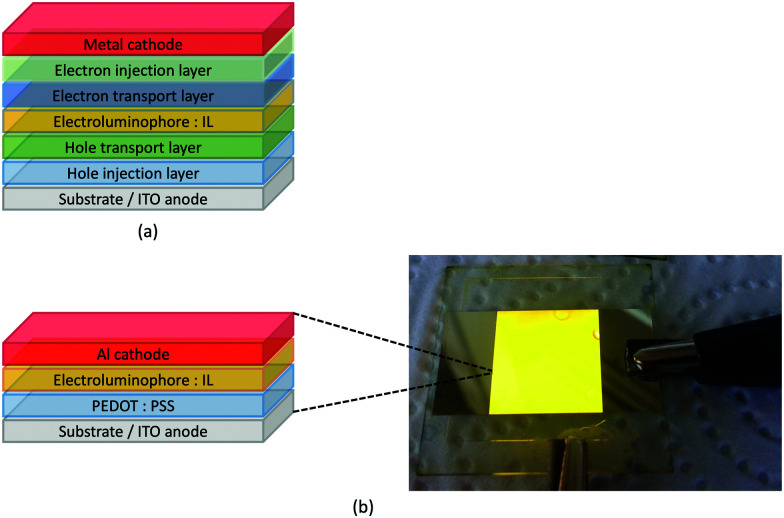
(a) Schematic representation of the layers in a typcial OLED; the cathode must consist of a metal with a low work function. (b) A working LEC (right) and a schematic illustration of a typical double-layer LEC. (ITO = indium tin oxide; PEDOT:PSS = poly(3,4-ethylenedioxythiophene):polystyrenesulfonate; IL = ionic liquid). Metals such as Ag or Au may replace Al as the cathode. In a single-layer LEC, the PEDOT:PSS hole injection layer is absent. In both devices, the substrate is usually glass. (Photo credit: Dr Collin Morris, University of Basel.).

LEDs and OLEDs are relatively complex multicomponent or multilayer devices (Fig. 1a), usually fabricated in expensive facilities operating at high temperatures and low pressures, and using potentially explosive and toxic materials. An alternative technology is to be found in the light-emitting electrochemical cell (LEC) which is phenomenologically related to the OLED, and which began to be seriously considered as a viable technology in the mid-1990s. Although LECs bear some relationship to OLEDs, they also exhibit important differences including a simpler device architecture (Fig. 1b), the possibility of routinely using low temperature solution-based fabrication techniques, and less restricted cathode materials allowing manufacture under ambient conditions.^4–8^

One fundamental distinction between OLEDs and LECs is the nature of the active (emissive) material: in an OLED this is typically a neutral species, whereas in a LEC, it is charged. However, the use of ionic transition metal complexes (iTMCs) in OLEDs is not excluded.^9,10^ Early LECs included luminescent polymers containing ionic salts.^11^ The first LEC in which an iTMC was used as the emissive species was reported in 1996 and contained a [Ru(bpy)_3_]^2+^-based material (bpy = 2,2′-bipyridine).^12^ However, the low stability of Ru-iTMC-containing LECs under operating conditions and the fact that the emission colour is invariably orange-red, limit the potential applications of such devices. The next family of iTMCs to be developed contained iridium(iii) coordination compounds, in particular [Ir(C^N)_2_(N^N)]^+^ complexes where C^N is a cyclometallated chelating ligand and N^N is a diimine or related chelating ligand. Advantages of iridium(iii) over ruthenium(ii) complexes lie in device stability and the ease of colour tuning the emission maxima.^4,13–17^ A disadvantage of iridium, however, is its low earth-abundance (*ca.* 3 × 10^−6^ ppm).^18^ With an earth's crustal abundance of *ca.* 50 ppm,^18^ copper is an attractive alternative to iridium, and a wide variety of copper(i) coordination compounds has been designed for applications as electroluminescent materials in both OLEDs and LECs.

### Why choose copper(i)?

The seminal work of McMillin and coworkers in the late 1970s and early 1980s laid the foundation for the development of copper(i) coordination compounds in LECs. In 1978, Buckner and McMillin reported that excitation into the metal-to-ligand charge transfer (MLCT) bands of the heteroleptic complexes [Cu(PPh_3_)_2_(bpy)]^+^ and [Cu(dpe)(bpy)]^+^ (dpe = (*E*)-bis(1,2-diphenylphosphano)ethene) led to photoluminescence (PL) originating from low-lying charge transfer excited states.^19^ This was followed by a series of investigations which demonstrated the emission behaviours of heteroleptic [Cu(P^P)(N^N)]^+^ and [Cu(PPh_3_)_2_(N^N)]^+^ and homoleptic [Cu(N^N)_2_]^+^ complexes in which P^P is a chelating bis(phosphane) and N^N is a bpy or phen-based ligand (phen = 1,10-phenanthroline).^20–26^ Of particular importance is the fact that [Cu(2,9-R_2_phen)_2_]^+^ (2,9-R_2_phen = 2,9-disubstituted-1,10-phenanthroline) were found to exhibit a long-lived emission in solution at room temperature.^23^ The photophysical properties of copper(i) complexes have been reviewed in detail,^27–29^ and we highlight several salient points that are especially relevant to the design of emissive materials for applications in LECs.

Copper(i) has a d^10^ configuration and four-coordinate copper(i) complexes are tetrahedral or distorted tetrahedral. Upon excitation, the metal centre is formally oxidized with a concomitant flattening of the coordination geometry towards the square-planar environment favoured by copper(ii). In the excited state, unless sufficiently protected by sterically demanding ligand-substituents, the Cu centre may be exposed to attack by nucleophiles including solvent molecules to give a five-coordinate exciplex. Steric hindrance resulting from the introduction of substituents into the 2,9-positions of phen in [Cu(phen)_2_]^+^ derivatives has a profound effect on the PL properties of the compounds.^27,30^ Tuning of the emission behaviour can also be achieved through other substituent effects,^31^ providing tremendous scope for ligand design. Upon going from homoleptic [Cu(N^N)_2_]^+^ to heteroleptic [Cu(P^P)(N^N)]^+^ complexes, emission behaviour is enhanced. This is a consequence of the reduced flexibility of the [Cu(P^P)(N^N)]^+^ coordination sphere which leads to a decrease in the non-radiative deactivation suffered by [Cu(N^N)_2_]^+^ coordination compounds.^32^ However, the solution PL of [Cu(P^P)(N^N)]^+^ species is highly dependent upon solvent and the presence of O_2_. An increase in photoluminescence quantum yield (PLQY) upon removal of O_2_ from a solution of a salt of [Cu(P^P)(N^N)]^+^ is a consequence of the suppression of exciplex quenching. The design of [Cu(P^P)(N^N)]^+^ and related emitters should, therefore, address appropriate steric shielding of the copper centre as we illustrate throughout this review.

Efficient emitters are essential for applications in LECs. In iTMC-LECs containing iridium(iii) complexes, the large spin–orbit coupling (SOC) of the third-row d-block metal leads to mixing of triplet and singlet states. After photoexcitation of an Ir-iTMC, fast intersystem crossing (ISC) from singlet to triplet excited states leads almost exclusively to spin-forbidden phosphorescence from the lowest triplet state (T_1_) to the ground-state S_0_.^16^ Since copper is in the first row of the d-block, SOC is small, and the mechansim described for Ir-iTMCs does not appertain. However, one of the most exciting prospects for copper(i) coordination compounds is their potential to exhibit thermally activated delayed fluorescence (TADF) in which the S_1_ and T_1_ excited states lie close in energy, permitting ISC (and reverse intersystem crossing, RISC) to occur without the requirement for a heavy metal.^33–36^

We should also note that in four-coordinate [Cu(P^P)(N^N)]^+^ complexes, the HOMO is typically largely located on the copper centre with some contribution from the phosphorus atoms, while the LUMO is localized on the N^N ligand.^37^ As in octahedral cyclometallated [Ir(ppy)_2_(bpy)]^+^ (Hppy = 2-phenylpyridine) derivatives,^16^ this spatial separation of the HOMO and LUMO character should allow for colour tuning of the emissions of heteroleptic copper(i) complexes. However, in practice, as we shall see later, this is less straightforward for copper(i) than for iridium(iii).

### Thermally activated delayed fluorescence

**Fig. 2:**
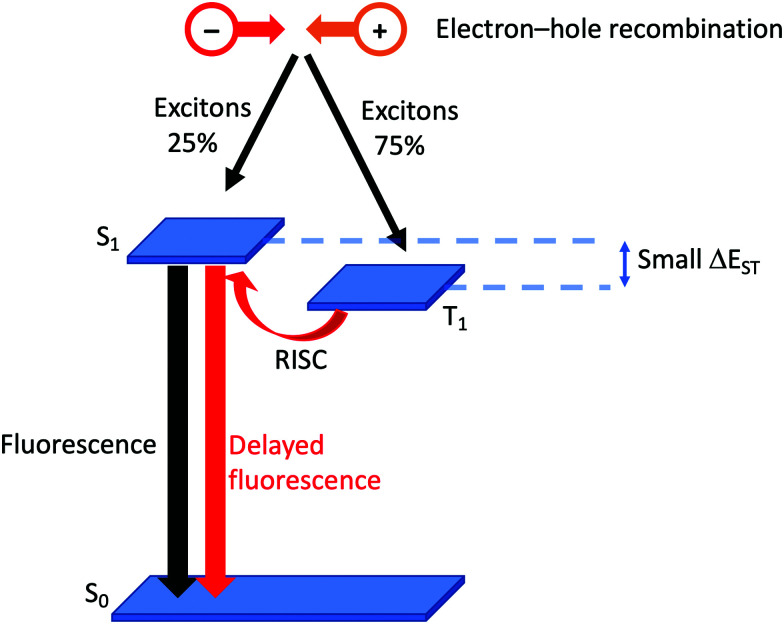
Schematic representation of TADF starting from the formation of excitons in a LEC. (RISC = reverse intersystem crossing).

Although it has been brought to the fore in respect of heteroleptic copper(i) coordination compounds over the last decade, the phenomenon of TADF has been known (although not originally by this acronym) since the 1960s.^38^ In compounds that display TADF, the energy gap between the singlet and triplet excited states, Δ*E*_ST_,††Values of the energy separation between the S_1_ and T_1_ excited states, Δ*E*_ST_, is given in either cm^−1^ or eV in the literature. For consistency, we have used both units, adding eV where the value is originally quoted in cm^−1^, and *vice versa*. is small (typically *ca.* 0.12 eV or 1000 cm^−1^).^35^ After photoexcitation (S_0_ → S_1_), ISC occurs and creates a triplet reservoir, the lifetime of which is long enough to allow reverse intersystem crossing, RISC, (T_1_ → S_1_) to occur. Repopulation of the singlet state leads to fluorescence (S_1_ → S_0_), and the TADF material is therefore classed as a singlet emitter. If Δ*E*_ST_ > *ca.* 3000 cm^−1^ (0.37 eV), thermal population of S_1_ is not effective.^39^ In a LEC or OLED, the recombination of electrons and holes leads to the formation of excitons in either a singlet (25%) or a triplet (75%) state (Fig. 2). If the decay of the triplet exciton is spin forbidden, the internal quantum efficiency is limited to 25%. As described above, the large SOC in Ir-iTMCs overcomes this limitation. On the other hand, the success and future exploitation of Cu-iTMCs depends upon TADF (Fig. 2).^33–35,40–46^

Experimentally, there are two qualitative probes to investigate TADF. The first is a comparison of the emission decay lifetimes, *τ*, at ambient and low (typically 77 K) temperatures. Longer lifetimes at 77 K compared to *ca.* 300 K are consistent with a T_1_ → S_0_ decay (phosphorescence) when RISC is not thermally accessible. The second is a red-shifting of the emission maxmium upon lowering the temperature. This corresponds to a change from predominant contributions to the emission from S_1_ → S_0_ (fluorescence at room temperature) to T_1_ → S_0_ (phosphorescence at low temperature). In addition to a small S_1_–T_1_ separation (Δ*E*_ST_, Fig. 2), characteristics of state-of-the-art TADF emitters are a high rate of fluorescence decay, a low rate of phosphoresence decay, fast RISC, and a short lifetime of the delayed fluorescence. For in depth discussions of TADF, with an emphasis on copper-containing species, readers are directed to the following references.^34–36,41,42,44–48^

## The early years

**Fig. 3:**
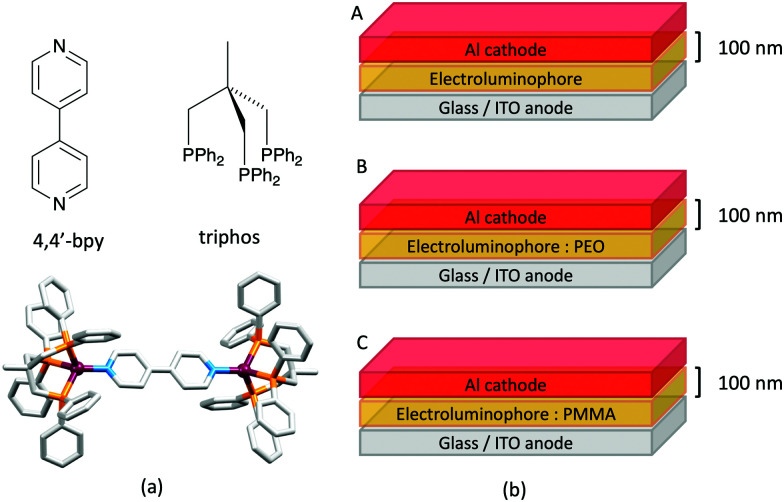
(a) Structures of 4,4′-bpy and triphos, and the structure of the [Cu_2_(triphos)_2_(μ-4,4′-bpy)]^2+^ cation in the [BF_4_]^−^ salt (CSD refcode BUPROV). (b) The three device architectures (A–C) used by Wang *et al.*^49^ PEO = poly(ethylene) oxide; PMMA = poly(methyl methacrylate). As a general note, 3D-structures in this review have been drawn using coordinates retrieved from the Cambridge Structural Database (CSD, version 2020.3.1)^50,51^ and using Mercury version 2020.3.1.^52^

Some of the original papers that uncovered the potential of heteroleptic copper(i) complexes for application in LECs were published before it had been fully recognized that TADF could be of importance in this class of compound. Although the synthesis, structure (Fig. 3a) and detailed photophysical properties of [Cu_2_(triphos)_2_(μ-4,4′-bpy)][BF_4_]_2_ were not published until 2015,^53^ this dinuclear complex was used as the electroluminphore in one-layer LECs as early as 2005.^49^ This appears to be the first report of a LEC based upon a copper(i)-based emitter. While maintaining a heteroleptic copper(i) core related to those of McMillin's complexes (see earlier discussion), the tripodal ligand triphos (Fig. 3) was selected for the rigidity that it would impose on the coordination sphere of the Cu(i) centre.^54^ In solution, [Cu_2_(triphos)_2_(μ-4,4′-bpy)][BF_4_]_2_ exhibits an absorption band at 366 nm assigned to MLCT,^49,53^ with a solid-state emission with *λ*^em^_max_(PL) = 555 nm^53^ and an excited state lifetime of 13.6 μs (298 K). Wang *et al.* fabricated LECs with three device architectures (Fig. 3b) with thin films of the emitting material spin-coated onto the glass/ITO anodes. PEO and PMMA are added to improve film formability (minimizing defects in the film) and stability. All devices had a low turn-on voltage (2 V for LEC in configuuration A, Fig. 3b) and exhibited red-orange emissions. The PL spectrum of the thin-film exhibited a maximum at 555 nm with a red shift of *ca.* 50 nm upon going to the electroluminesence (EL) spectrum. Under forward bias, the EL maximum was 590 nm, and under reverse bias, *λ*^em^_max_(EL) = 618 nm. The origins of this red-shift were attributed to polarization effects and appear analogous to those observed by Slinker *et al.* for LECs based upon Ir-iTMCs.^55^ Device C (Fig. 3b) showed a maximum current efficiency of 0.16 cd A^−1^ when the LEC was driven at 12 V.

**Scheme 1:**
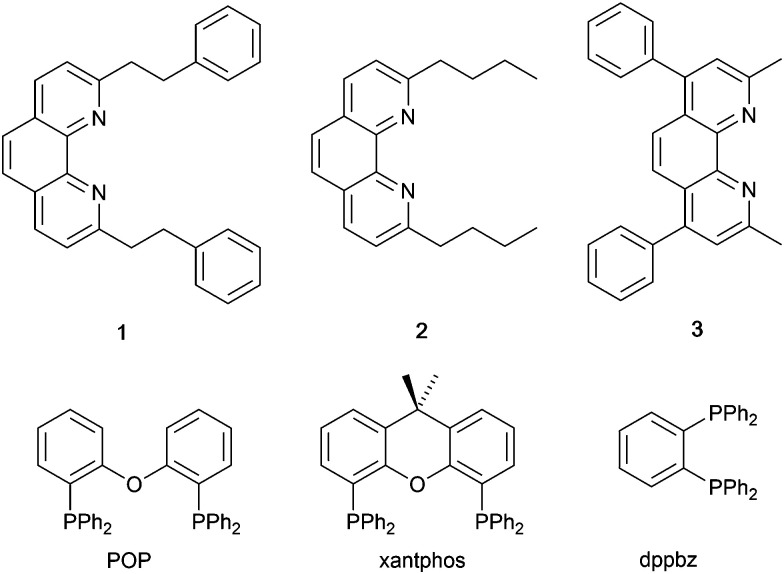
Structures of 1,10-phenanthroline derivatives 1–3, and the bis(phosphanes) POP, xantphos and dppbz.

**Fig. 4:**
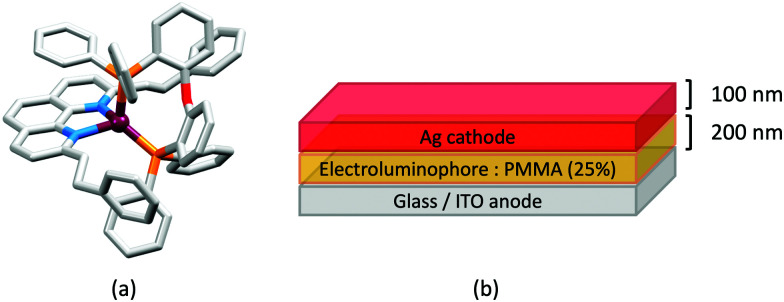
(a) The structure of the [Cu(POP)(1)]^+^ cation in [Cu(POP)(1)][BF_4_] (CSD refcode GIRJUN) showing the distorted tetrahedral Cu(i) environment and wide-bite angle (117.98(3)°) of POP (H atoms are omitted). The phen backbone is rigid (N–Cu–N = 80.86(10)°). (b) LEC architecture for a device containing [Cu(POP)(2)][BF_4_] as the electroluminophore.

In 2006, Armaroli reported the first application of mononuclear [Cu(P^P)(N^N)]^+^ emitters in LECs. The series of complexes incorporated the phen derivatives 1–3 with the bis(phosphane) POP (Scheme 1). The phen ligands contain 2,9-substituents to prevent flattening of the coordination sphere upon excitation (see earlier discussion) and we will see this feature repeatedly in N^N ligand design. POP and xantphos (Scheme 1) appear in many of the heteroleptic copper(i) complexes described in this review. Both are wide-bite angle chelating ligands,^56^ and are commercially available. The POP backbone is conformationally flexible and for 284 crystal structures in the CSD (version 2020.3.1)^50,51^ containing 326 independent chelating {Cu(POP)} domains, the P–Cu–P angle ranges from 103.08 to 121.78° with a mean value of 113.90°. The single-crystal structure of [Cu(POP)(1)][BF_4_] (Fig. 4a) confirms a P–Cu–P angle of 117.98(3)°. The Cu(i) centre is in a distorted tetahedral environment and the Cu⋯O distance of 3.226(2) Å is outside bonding range. Correlations of solid-state PLQY and Cu⋯O distances demonstrate a general trend for increased PLQY values with longer Cu⋯O separations,^57^ and therefore the note by Armaroli *et al.* in 2006^58^ that [Cu(POP)(1)]^+^ exhibits a long Cu⋯O separation is significant. In deaerated CH_2_Cl_2_ solutions, [Cu(POP)(1)][BF_4_], [Cu(POP)(2)][BF_4_] and [Cu(POP)(3)][BF_4_] show emission maxima between 544 and 558 nm with PLQY values of 9, 26 and 27%, respectively. In 2006, these latter values were the highest reported for luminescent [Cu(P^P)(N^N)]^+^ complexes incorporating phen-derivatives. Single-layer LECs with [Cu(POP)(2)][BF_4_] mixed wth PMMA in the active layer (Fig. 4b) showed moderate efficiency, emitting green light after several minutes under 18 V bias. Notably, though, the performance was comparable to LECs based on [Ru(bpy)_3_][PF_6_]_2_ electroluminophores.^58^

**Fig. 5:**
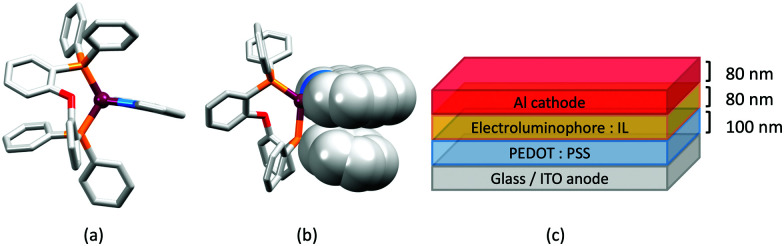
Structures of the complex cations (H atoms omitted) in (a) [Cu(POP)(bpy)][PF_6_] and (b) [Cu(POP)(phen)][PF_6_] (CSD refcodes OYUKID and OYUKUP). The face-to-face π-stacking interaction in [Cu(POP)(phen)]^+^ is shown in space-filling representation. (c) Architecture of the LEC devices containing [Cu(P^P)(N^N)][PF_6_] with N^N = bpy or phen, and P^P = POP or dppbz. The ionic liquid (IL) was [BMIM][PF_6_] or [EMIM][PF_6_], and Cu-iTMC: IL molar ratios was 1 : 1 or 1 : 0.

No further progress was made with Cu-iTMCs until our own report in 2011 which demonstrated the potential for bpy-containing heteroleptic compounds in LECs.^59^ We look at this study in some detail because of several general points which emerge that are relevant to later investigations. Comparisons of the single-crystal structures of [Cu(P^P)(N^N)][PF_6_] with N^N = bpy or phen, and P^P = POP or dppbz (Scheme 1) revealed several notable features. The P–Cu–P angles of 115.01(2)° in [Cu(POP)(bpy)]^+^ (Fig. 5a) and 119.18(2)° in [Cu(POP)(phen)]^+^ (Fig. 5b) are significantly greater than in the corresponding dppbz complex cations (92.50(2) and 87.14(8)°). In [Cu(POP)(phen)]^+^, one phenyl ring of a PPh_2_ unit in POP engages in a π-stacking interaction with the phen ligand (Fig. 5b). Such interactions help to lock the molecular geometry, and contribute to increased PLQY.^60^ Inspection of Fig. 5a reveals the potential for a face-to-face π-interaction between one phenyl ring of a PPh_2_ unit and one arene ring of the POP backbone. While the metrics of this interaction in [Cu(POP)(bpy)]^+^ are not consistent with an efficient interaction, similar contacts are a recurring feature in [Cu(POP)(N^N)]^+^ complexes, and indeed in other POP-containing compounds. We highlight the importance of intra-cation π-stacking interactions throughout this review.

Typical broad MLCT bands between 389 and 422 nm were observed in the absorption spectra of [Cu(POP)(bpy)][PF_6_], [Cu(POP)(phen)][PF_6_], [Cu(dppbz)(bpy)][PF_6_] and [Cu(dppbz)(phen)][PF_6_]. Analysis of the emission behaviour was supplemented by density functional theory (DFT) calculations which confirmed the ^3^MLCT character of the lowest tripet excited state (^3^T_1_). The calculated values of the vertical energy difference between the ^3^T_1_ and S_0_ levels were in good agreement with the experimental values for *λ*^em^_max_(PL) which were in the range 648–662 nm in solution and 599–610 nm for thin films having the same composition (Cu-iTMC: ionic liquid [EMIM][PF_6_] molar ratio = 1 : 1, [EMIM][PF_6_] = 1-ethyl-3-methylimidazolium hexafluoridophosphate) as those used in LECs. The PLQY values for deaerated solutions of the Cu-iTMCs were <1%, and ranged from 1.0–9.0% for thin films, the highest being for [Cu(POP)(phen)][PF_6_]. The architectures of the LECs (Fig. 5c) differed from those shown in Fig. 3 and 4 by incorporating a PEDOT:PSS hole-injection layer. Additionally, the Cu-iTMC was mixed with an ionic liquid (IL) in order to improve device performance by increasing the ionic conductivity of the active layer. Both [EMIM][PF_6_] and [BMIM][PF_6_] ([BMIM][PF_6_] = 1-butyl-3-methylimidazolium hexafluoridophosphate) were used, with the former proving to be more beneficial. Device performances compared favourably with many based on Ru-iTMCs or Ir-iTMCs, with LECs based on [Cu(POP)(bpy)][PF_6_] and [Cu(POP)(phen)][PF_6_] exhibiting efficiencies of 1.64 cd A^−1^ under a 4 V bias, and 4.55 cd A^−1^ under a 3 V bias, respectively. Luminance levels were greater using higher biases but this improvement came at the expense of device stability and efficiency. Significantly, our later studies in 2018 confirmed that [Cu(POP)(bpy)][PF_6_] is indeed a TADF emitter.^61^

**Fig. 6:**
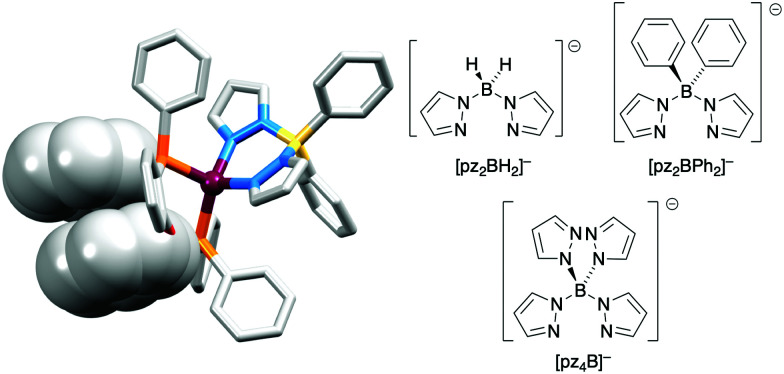
The structure of [Cu(POP)(pz_2_BPh_2_)] (CSD refcode YACMIA) with the π-stacking interaction in POP highlighted (H atoms omitted), and the structures of the N^N ligands in [Cu(POP)(pz_2_BH_2_)], [Cu(POP)(pz_2_BPh_2_)] and [Cu(POP)(pz_4_B)].

The realization that heteroleptic Cu(i) complexes could exhibit TADF seems first to have come with an investigation of the neutral compounds [Cu(POP)(pz_2_BH_2_)], [Cu(POP)(pz_2_BPh_2_)] and [Cu(POP)(pz_4_B)] (see Fig. 6 for the N^N ligand structures). Being neutral, these compounds are more relevant to OLEDs than LECs. However, these ground-breaking results from Yersin and coworkers^62^ came at a pivotal point in the development of Cu-iTMCs for LECs. [Cu(POP)(pz_2_BH_2_)], [Cu(POP)(pz_2_BPh_2_)] and [Cu(POP)(pz_4_B)] contain distorted tetrahedral Cu(i) centres and the conformation of each coordinated POP ligand facilitates an intra-ligand π-stacking interaction (Fig. 6a). The compounds are strongly emissive with solid-state PLQYs of 45, 90 and 90%, respectively. Below 100 K, the luminescence originates from the T_1_ state; at 1.6 K, the emission bands are broad and unstructured with *λ*^em^_max_(PL) values lying between 453 and 474 nm for the three compounds, and decay times (30–100 K) in the range 450 to 610 μs. Above 100 K, the emissions are blue-shifted and the decay times are shorter, consistent with emission from the lowest excited singlet state S_1_. This is the dominant emission at room temperature. For [Cu(POP)(pz_2_BH_2_)], [Cu(POP)(pz_2_BPh_2_)] and [Cu(POP)(pz_4_B)], the S_1_–T_1_ separation (Δ*E*_ST_, Fig. 2) was determined to be 1300, 800 and 1000 cm^−1^ (0.16, 0.10 and 0.12 eV), *i.e.* small enough to allow TADF to occur.

**Scheme 2:**
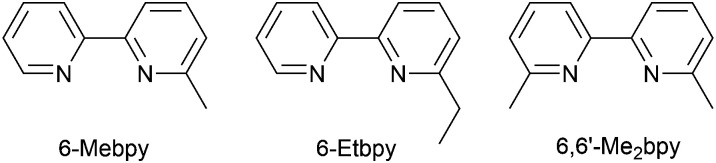
Structures of the ligands 6-methyl- and 6-ethyl-2,2′-bipyridine, and 6,6′-dimethyl-2,2′-bipyridine.

**Fig. 7:**
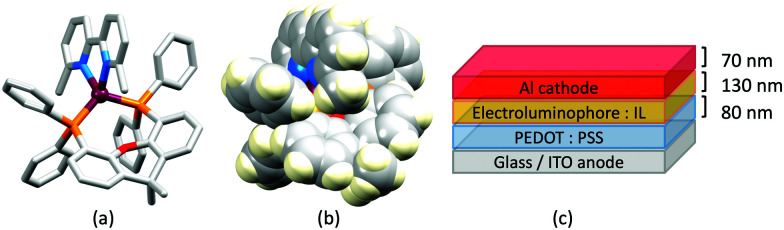
The structure of the [Cu(xantphos)(6,6′-Me_2_bpy)]^+^ cation in the [PF_6_]^−^ salt (CSD refcode GABVAJ): (a) with H atoms omitted, and (b) in space-filling representation to show the steric shielding of the Cu(i) centre. (c) Architecture of the LEC devices containing [Cu(xantphos)(N^N)][PF_6_] with N^N = 6-Mebpy, 6-Etbpy or 6,6′-Me_2_bpy. IL = [EMIM][PF_6_], and Cu-iTMC: IL molar ratio = 4 : 1.

In the immediate years after Yersin and coworkers' findings,^62^ the relevance of TADF to the emission behaviour of heteroleptic copper(i) coordination compounds and promising performances in LECs began to be appreciated, although it is important to note that measurements of low temperature emission spectra were not routinely carried out to confirm the TADF phenomenon. Thus, in some cases, Cu-iTMCs were not described as TADF emitters even though the phenomenon may have been, or was, operative. For example, we developed a series of [Cu(POP)(N^N)][PF_6_] and [Cu(xantphos)(N^N)][PF_6_] compounds in which N^N was a 6-alkyl- or 6,6′-dialkyl-2,2′-bipyridine (Scheme 2).^37,63^ The emissive properties of these complexes were enhanced with respect to those of [Cu(POP)(bpy)][PF_6_] (see above), consistent with the expectations of introducing sterically demanding substituents close to the Cu(i) centre (Fig. 7a and b). Among this series of compounds, the highest PLQYs were observed for solid-state [Cu(xantphos)(6-Mebpy)][PF_6_] (34%), [Cu(xantphos)(6-Etbpy)][PF_6_] (37%) and [Cu(xantphos)(6,6′-Me_2_bpy)][PF_6_] (37%) with lifetimes of 9.6–11 μs. DFT calculations predicted that the emitting T_1_ state involved ^3^MLCT character. Significantly, we found that the S_1_–T_1_ energy difference was in the range of 0.17–0.21 eV (*ca.* 1400–1700 cm^−1^) for all the complexes in the series. We noted that, while this was larger than the value of Δ*E*_ST_ proposed by Leitl *et al.*^39^ to allow population of the S_1_ from T_1_ at 298 K, contributions to the room temperature emission from fluorescence (S_1_ → S_0_) could not be discounted.^37^ LECs with the architecture shown in Fig. 7c were operated under a pulsed current, and exhibited rapid turn-on times. The LEC with [Cu(xantphos)(Me_2_bpy)][PF_6_] in the active layer (*λ*^em^_max_(EL) = 567 nm) achieved a maximum efficacy of 3.0 cd A^−1^ and a luminance of (145 cd m^−2^) with a device lifetime of 1 hour. In contrast, the lifetimes of LECs containing [Cu(xantphos)(Mebpy)][PF_6_], [Cu(xantphos)(6-Etbpy)][PF_6_] and [Cu(POP)(6-Etbpy)][PF_6_] were greater than 15, 40 and 80 hours, respectively, but at a cost of lower efficacy (1.9, 1.7 and 0.6 cd A^−1^).

## The TADF era in Cu-iTMCs begins

**Scheme 3:**
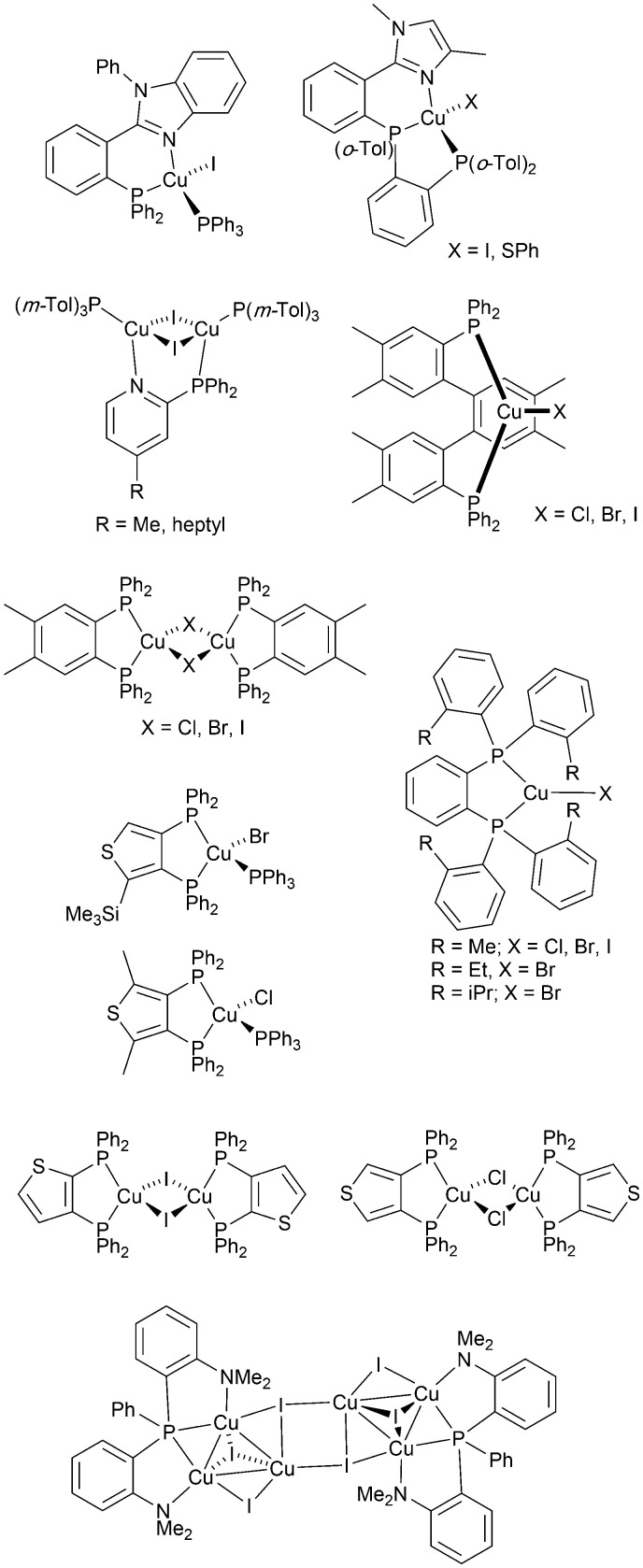
Structures of selected neutral, TADF copper(i) complexes with Cu–X bonds which have been tested in OLEDs.

From 2012 onwards, reports of Cu-iTMCs exhibiting TADF grew considerably, although not all compounds have been tested in LECs. Heteroleptic copper(i) compounds for which TADF has been described include halide and pseudo-halide containing complexes including several {Cu_*x*_X_*y*_} clusters. Most of these are neutral and are of interest for OLEDs; selected examples which have been tested in OLED configurations are shown in Scheme 3.^36,64–96^

Copper(i) coordination compounds that class as Cu-iTMCs include those containing four-coordinate [Cu(P^P)(N^N)]^+^, [Cu(P)_2_(N^N)]^+^, [Cu(P)(tripodal-N_3_)]^+^, [Cu(P)(N^N)(N)]^+^, [Cu(P^P)(N^S)]^+^, [Cu(P^P)(P^S)]^+^, [Cu(P^P)(NHC)]^+^ (NHC = N-heterocyclic carbene) coordination domains, dinuclear complexes with P^P and N^N ligands, three-coordinate [Cu(N^N)(NHC)]^+^ and two-coordinate [Cu(N)(NHC)]^+^ complexes. We now consider emissive materials in each class, and provide insight into design of appropriate ligands and ligand combinations to enhance photoluminescence. In terms of applications in LECs, we note that efficient PL is not necesssarily an indication that a Cu-iTMC will perform well as an electroluminophore in a device. Selected neutral compounds with structures related to those in the classes of Cu-iTMCs but which have been designed for OLED applications are also included in our discussion.

## Mononuclear [Cu(P^P)(N^N)]^+^ and [Cu(P)_2_(N^N)]^+^

### 2,2′-Bipyridine derivatives with POP and xantphos

**Scheme 4:**
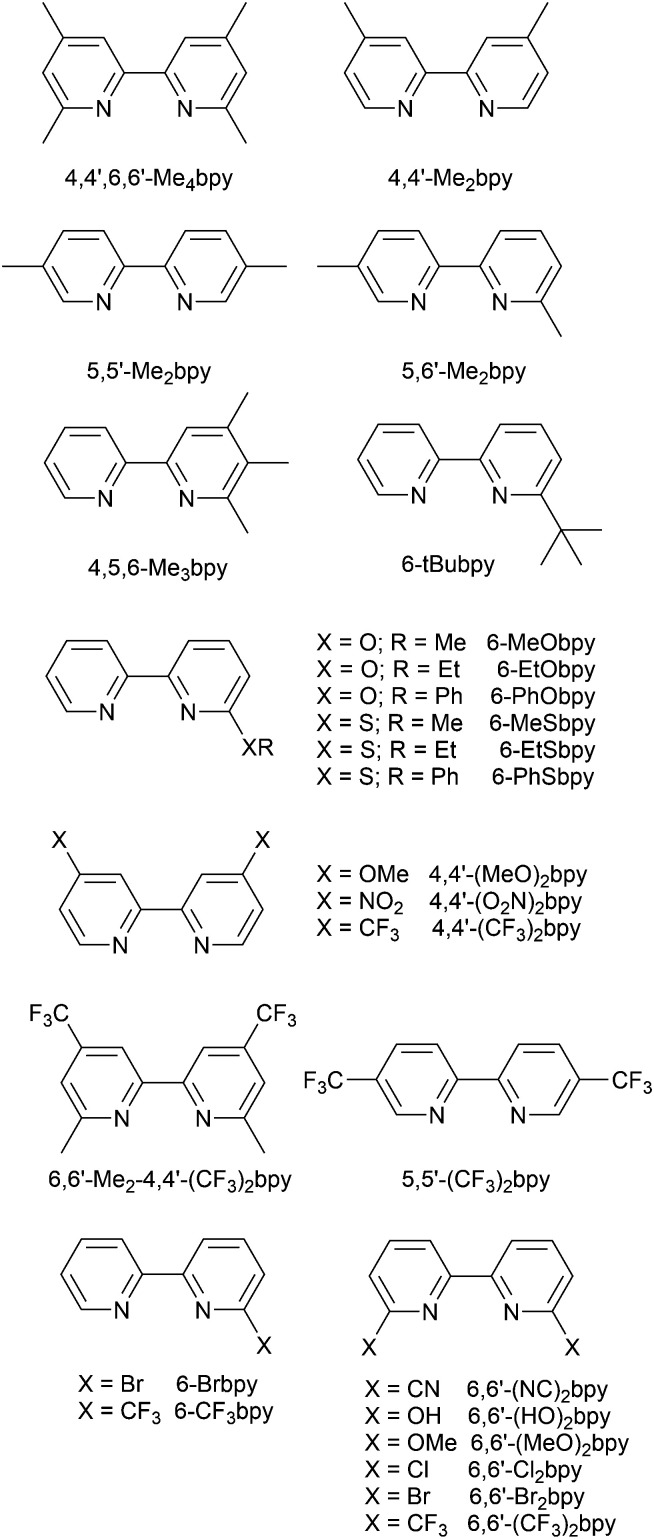
Structures of derivatives of bpy used in [Cu(P^P)(N^N)]^+^ complexes. See also Scheme 2.

**Fig. 8:**
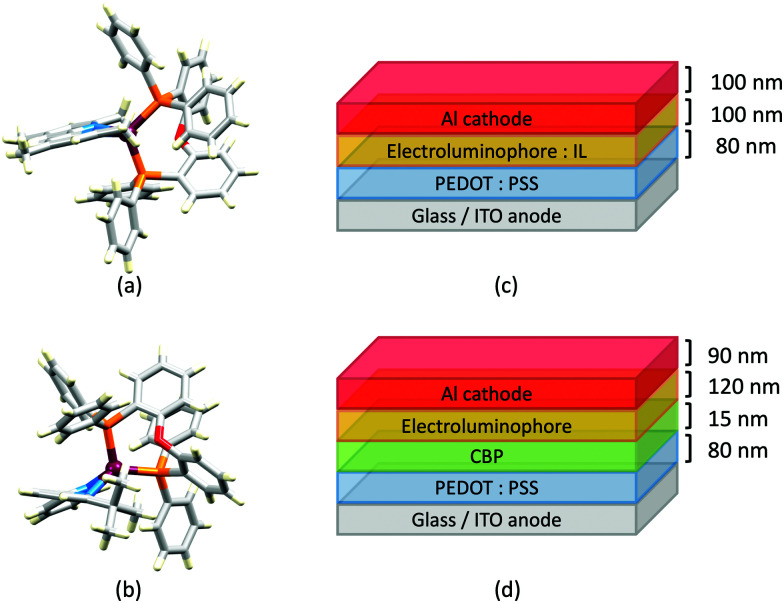
Structures of the cations in (a) [Cu(POP)(4,4′,6,6′-Me_4_bpy)][BF_4_] (CSD refcode COYHEF), and (b) [Cu(POP)(6-*t*Bubpy)][PF_6_] (CSD refcode PUTSUV). (c) Architecture of the LECs containing the Cu-iTMCs shown in Table 3. IL = [EMIM][PF_6_], and Cu-iTMC: IL molar ratio = 4 : 1; LECs were driven using a pulsed current. The layer thicknesses shown apply to devices reported in ref. 98, and are typical. (d) Bilayer LEC architecture used for devices containing [Cu(POP)(6,6′-(MeO)_2_bpy)][PF_6_] in the active layer (CBP = 4,4′-bis(9-carbazolyl)-1,1′-biphenyl).

Scheme 4 illustrates the structures of the N^N ligands discussed in this section. Around the same time that we were investigating the performances of LECs containing [Cu(POP)(6-Mebpy)][PF_6_] or [Cu(POP)(6,6′-Me_2_bpy)][PF_6]_,^63^ Yersin, Robertson and coworkers compared the photophysical properties of [Cu(POP)(4,4′-Me_2_bpy)][BF_4_] and [Cu(POP)(4,4′,6,6′-Me_4_bpy)][BF_4_]. They demonstrated that the presence of the 6,6′-substituents led to a dramatic increase in room temperature solid-state PLQY from 9% for [Cu(POP)(4,4′-Me_2_bpy)][BF_4_] to 55% or 74% (enhanced when the sample was ground) for [Cu(POP)(4,4′,6,6′-Me_4_bpy)][BF_4_].^32^ These results are in accord with our findings that the solid-state PLQY of 43.2% for [Cu(POP)(6,6′-Me_2_bpy)][PF_6_] exceeds that of [Cu(POP)(6-Mebpy)][PF_6_] (9.5%)^63^ and [Cu(POP)(bpy)][PF_6_] (3%).^97^ The emission spectrum of a powdered sample of [Cu(POP)(4,4′,6,6′-Me_4_bpy)][BF_4_] (*λ*_exc_ = 350 nm) is broad at 300 K with *λ*^em^_max_(PL) = 555 nm and a decay time of 11 μs. On cooling to 77 K, the emission undergoes a red-shift to 575 nm with PLQY = 47%, and the decay time increases to 87 μs. The ≈10-fold increase in the radiative rate on going from 77 to 300 K, coupled with the blue-shift from 575 to 555 nm, were rationalized in terms of TADF at 300 K (S_1_ → S_0_ emission), while at 77 K, emission occurs from the T_1_ state (T_1_ → S_0_). In addition, Yersin and Robertson also confirmed that the restricted flexibility of the Cu coordination sphere caused by the presence of the 6,6′-dimethyl groups in the N^N ligand (Fig. 8a) resulted in a decrease in non-radiative deactivation with a consequent increase of PLQY.^32^ A comparison of PL behaviour of [Cu(POP)(Me_n_bpy)]^+^ complexes in which Me_n_bpy carries different numbers of Me substituents in differing positions (Table 1) gives a clear conclusion: substitution at the 6-position or 6,6′-positions is essential for high PLQY values (Table 1). Similar trends are seen for analogous xantphos-containing compounds (Table 1), and the steric shielding of the Cu(i) centre in [Cu(xantphos)(6,6′-Me_2_bpy)][PF_6_] was shown in Fig. 7b. However, the observation of Linfoot *et al.* that the PLQY of powdered [Cu(POP)(4,4′,6,6′-Me_4_bpy)][BF_4_] depends upon the morphology of the sample^32^ leads us to be cautious about further detailed interpretation of the PLQY data.

**Table 1:** Room temperature PL emission maxima, PLQY values and decay lifetimes (*τ*) for solid-state [Cu(POP)(Me_*n*_bpy)]^+^ and [Cu(xantphos)(Me_*n*_bpy)]^+^ complexes

Complex cation	*λ* ^em^_max_ (*λ*_exc_)/nm	PLQY/%	*τ*/μs	Ref.
[Cu(POP)(bpy)]^+^ a	580 (390)	3.0	1.5	97
[Cu(POP)(6-Mebpy)]^+^ a	567 (365)	9.5	2.6^c^	63
[Cu(POP)(6,6′-Me_2_bpy)]^+^ a	535 (365)	43.2	10.5^c^	63
[Cu(POP)(5,5′-Me_2_bpy)]^+^ a	585 (365)	2.7	2.3	98
[Cu(POP)(4,4′-Me_2_bpy)]^+^ b	575 (350)	9	-	32
[Cu(POP)(5,6′-Me_2_bpy)]^+^ a	553 (365)	12	6^c^	99
[Cu(POP)(4,5,6-Me_3_bpy)]^+^ a	518 (365)	42.7	9.3	98
[Cu(POP)(4,4′,6,6′-bpy)]^+^ b	555 (350)	55 (74)^d^	11 (13)^d^	32
[Cu(xantphos)(bpy)]^+^ a	587 (390)	1.7	1.3	97
[Cu(xantphos)(6-Mebpy)]^+^ a	547 (365)	33.8	9.7	37
[Cu(xantphos)(6,6′-Me_2_bpy)]^+^ a	539 (365)	37.3	11.4	37
[Cu(xantphos)(5,5′-Me_2_bpy)]^+^ a	571 (365)	6.3	5.1	98
[Cu(xantphos)(5,6′-Me_2_bpy)]^+^ a	555 (365)	11	5^c^	99
[Cu(xantphos)(4,5,6-Me_3_bpy)]^+^ a	529 (365)	58.8	9.8	98

a [PF_6_]^−^ salt.

b [BF_4_]^−^ salt.

c *τ* from a biexponential fit (see original work for details).

d The higher value is for a ground sample.

For some of the compounds in Table 1, emission data at 77 K have been reported and are presented in Table 2. In all cases, the longer decay time at 77 K *vs.* than at 300 K is consistent with TADF at ambient temperatures, even for complexes containing the parent bpy ligand. The second effect is a red-shift in the emission (compare *λ*^em^_max_(PL) at *ca.* 300 K in Table 1 with values at 77K in Table 2). For the compounds in Table 2, calculated values of Δ*E*_ST_ are between 0.22 and 0.27 eV (*ca.*, 1800–2200 cm^−1^). The introduction of a *tert*-butyl substituent into the 6-position of bpy (Scheme 4) leads to a decrease in Δ*E*_ST_ from 0.15 eV (1200 cm^−1^) for [Cu(POP)(6-*t*Bubpy)]^+^ to 0.17 eV (1370 cm^−1^) for [Cu(xantphos)(6-*t*Bubpy)]^+^. However, the steric demands of the *tert*-butyl group cause significant elongation of the Cu–N bonds (*ca.* 0.26 Å longer than is typical), and the N–C–C–N torsion angle of the bpy unit (−28.8(8)°) is noticeably larger in [Cu(POP)(6-*t*Bubpy)][PF_6_] (Fig. 8b) than in related compounds. These structural factors are likely to enhance raditionless decay from the T_1_ state with concomitant reduced emission. The room temperature solid-state PLQYs of [Cu(POP)(6-*t*Bubpy)][PF_6_] and [Cu(xantphos)(6-*t*Bubpy)][PF_6_] are 1.1 and 9.6%, and *τ* = 0.4 and 3.3 μs, respectively, values that are significantly lower than many of the methyl-substituted derivatives in Table 1.^98^

**Table 2:** Photoluminescence emission maxima and decay lifetimes (*τ*) for solid-state [Cu(POP)(Me_*n*_bpy)]^+^ and [Cu(xantphos)(Me_*n*_bpy)]^+^ complexes at 77 K

Complex cation	*λ* ^em^_max_(*λ*_exc_)/nm	*τ*/μs	Ref.
[Cu(POP)(bpy)]^+^ a	610 (410)	16	97*a*
[Cu(POP)(5,5′-Me_2_bpy)]^+^ a	591 (410)	63	98
[Cu(POP)(4,5,6-Me_3_bpy)]^+^ a	566 (410)	81	98
[Cu(POP)(4,4′,6,6′-bpy)]^+^ b	575 (378)	87	32
[Cu(xantphos)(bpy)]^+^ a	613 (410)	11	97*a*
[Cu(xantphos)(5,5′-Me_2_bpy)]^+^ a	594 (410)	44	98
[Cu(xantphos)(4,5,6-Me_3_bpy)]^+^ a	559 (410)	75	98

a [PF_6_]^−^ salt.

b [BF_4_]^−^ salt.

Some of the best performing copper-based LECs have been achieved using electroluminophores comprising [Cu(POP)(N^N)][PF_6_] salts in which N^N is a simple derivative of bpy (Table 3). Most noteworthy is a LEC containing [Cu(xantphos)(4,5,6-Me_3_bpy)][PF_6_] with the device architecture shown in Fig. 8c. This reached a maximum luminance of 462 cd m^−2^ and exhibited a device half-life of up to 98 hours. However, turn-on times are typically of the order of minutes or hours (Table 3). Faster turn-on times have been observed for LECs containing [Cu(xantphos)(6-Phbpy)][PF_6_] (6-Phbpy = 6-phenyl-2,2′-bipyridine), but this is at the expense of luminance (Lum_max_ = 5 cd m^−2^).^37^ Included in Table 3 is a LEC containing [Cu(POP)(6-EtObpy)][PF_6_]. This is one of a series of [Cu(POP)(N^N)][PF_6_] and [Cu(xantphos)(N^N)][PF_6_] Cu-iTMCs incorporating 6-RObpy or 6-RSbpy ligands (R = Me, Et, Ph, see Scheme 4) which are yellow emitters. Powdered samples have PLQYs up to 38%, with emission lifetimes ≤10.2 μs at *ca.* 298 K. Lifetimes are extended to between 11 and 48 μs at 77 K, consistent with TADF at ambient temperatures. A noteworthy feature of the LEC with [Cu(POP)(6-EtObpy)][PF_6_] in the emitting layer was the relatively long device lifetime; the time for the EL to decay to half the maximum luminance was 200 hours.^100^ The electron-donating properties of the MeO substituents have also been exploited by Barolo, Costa and coworkers in LECs containing [Cu(POP)(6,6′-(MeO)_2_bpy)][PF_6_] in the emitting layer. Powdered [Cu(POP)(6,6′-(MeO)_2_bpy)][PF_6_] has a PLQY of 14%, and in thin-film, this increases to 20% (*λ*_exc_ = 370 nm); TADF behaviour was not investigated. However, in terms of this review, this work is noteworthy for a change in LEC design aimed at minimizing the irreversible formation of Cu(ii) species. By using the bilayer LEC architecture shown in Fig. 8d, the goal was to decouple hole/electron injection and transport.^101^

**Table 3:** Electroluminescence maxima and LEC performances (architectures as in Fig. 8c) measured using pulsed current driving. All complexes are [PF_6_]^−^ salts

Complex cation	*λ* ^em^_max_/nm	*J*_avg_/A m^−2^ a	*t*_on_/min b	Lum_max_/cd m^−2^	Ref.
[Cu(POP)(4,5,6-Me_3_bpy)]^+^	571	50	11	92	98
[Cu(xantphos)(4,5,6-Me_3_bpy)]^+^	570	100	13	462	98
[Cu(xantphos)(5,5′-Me_2_bpy)]^+^	589	100	19	130	98
[Cu(POP)(6-EtObpy)]^+^	585	50	60	63	100
[Cu(xantphos)(6-CF_3_bpy)]^+^	589	100	137	109	61
[Cu(POP)(2-Etphen)]^+^	582	100	25	451	98
[Cu(xantphos)(2-Etphen)]^+^	580	100	122	153	98

a *J*_avg_ = average current density.

b Time to reach maximum luminance (Lum_max_).

**Fig. 9:**
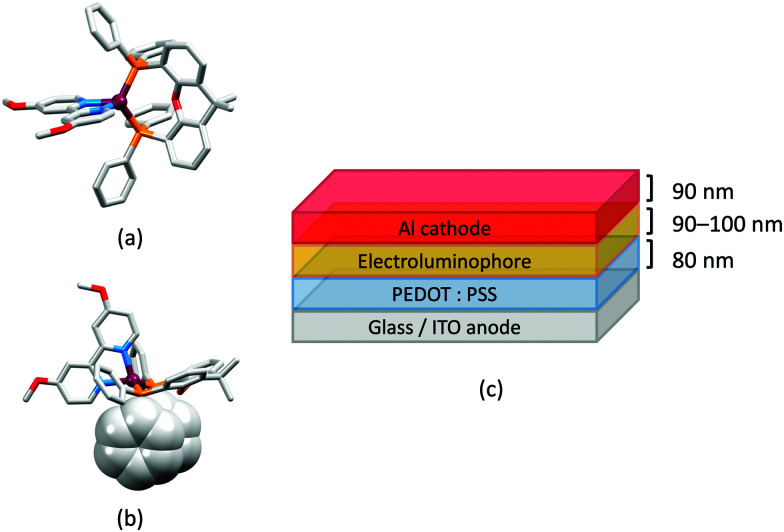
(a) Structure of the cation in [Cu(xantphos)(4,4′-(MeO)_2_bpy)][BF_4_] (CSD refcode VANYOB), and (b) illustration of the π-stacking interaction between adjacent PPh_2_ units. H atoms are omitted for clarity. (c) Architecture of the LECs containing the Cu-iTMCs [Cu(xantphos)(4,4′-R_2_bpy)][BF_4_] with R = MeO, Me, H. LECs were driven using a pulsed current.

In 2017, Weber *et al.* published the results of an informative investigation correlating the effect of the σ-Hammett parameter, *σ*_p_, of the substituents in the N^N ligands 4,4′-Me_2_bpy, 4,4′-(MeO)_2_bpy, bpy and 4,4′-(O_2_N)_2_bpy (Scheme 4) on the PL and EL properties of [Cu(xantphos)(N^N)][BF_4_]. The presence of MeO groups (with the most negative *σ*_p_) leads to the highest solid-state PLQY (18.9% compared to 9.7% for N^N = 4,4′-Me_2_bpy, 0.51% for unsubstituted bpy, and no emission for N^N = 4,4′-(O_2_N)_2_bpy). The latter is attributed to the different nature of the lowest excited state of [Cu(xantphos)(4,4′-(O_2_N)_2_bpy)]^+^ compared to that of the other members of this series of Cu-iTMCs. The absorption spectrum of [Cu(xantphos)(4,4′-(O_2_N)_2_bpy)][BF_4_] exhibits a broad band centred at 503 nm with a shoulder at 423 nm (not present in compounds with 4,4′-Me_2_bpy, 4,4′-(MeO)_2_bpy and bpy), and these are assigned to combinations of d–d, MLCT and intra-ligand transitions. There is a linear relationship between the *σ*_p_ values of the 4,4′-substituents in the bpy ligand and the values of *λ*^em^_max_(PL) for solid [Cu(xantphos)(N^N)][BF_4_] (*λ*_exc_ = 376 nm): 545 nm for N^N = 4,4′-(MeO)_2_bpy, 570 nm for 4,4′-Me_2_bpy, and 600 nm for bpy.^102^ This latter value compares with 587 nm reported for powdered [Cu(xantphos)(bpy)][PF_6_] (*λ*_exc_ = 365 nm).^61^ Critically, Weber *et al.* note that the TADF effect in the Cu-iTMCs may depend upon the *σ*_p_ value of the 4,4′-substituents in the bpy ligand. The structure of the [Cu(xantphos)(4,4′-(MeO)_2_bpy)]^+^ cation is depicted in Fig. 9a and b and it is worth noting that the π-stacking interaction between two phenyl rings of different PPh_2_ units is a common feaure in [Cu(xantphos)(N^N)]^+^ complexes. Testing of LECs with the architecture shown in Fig. 9c and with [Cu(xantphos)(N^N)][BF_4_] (N^N = 4,4′-Me_2_bpy, 4,4′-(MeO)_2_bpy, bpy and 4,4′-(O_2_N)_2_bpy) in the active layer (no IL was added) led to the conclusions that (i) nitro groups (positive *σ*_p_) gave no EL even though charge injection occurred, and (ii) methoxy groups (most negative *σ*_p_) resulted in the highest luminance (54 cd m^−2^) and the most stable devices.^102^ The correlations established in this work may provide a basis for further development of structure–property relationships in Cu-iTMCs.

**Fig. 10:**
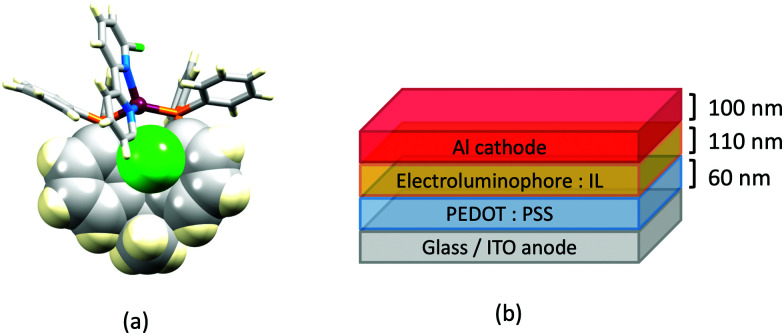
(a) Structure of the cation in [Cu(xantphos)(6,6′-Cl_2_bpy)][PF_6_] (CSD refcode MEWXUK). (b) Architecture of LECs containing the Cu-iTMCs with halogen-substituted bpy ligands. IL = [EMIM][PF_6_] and Cu-iTMC: IL molar ratio = 4 : 1. LECs were driven using a pulsed current.

The effects of electron-withdrawing (CN, Cl, Br, CF_3_) and electron-donating (OH) groups in the 6- and 6,6′-positions of bpy (Scheme 4) on the PL and EL properties of [Cu(P)_2_(N^N)]^+^ and [Cu(P^P)(N^N)]^+^ complexes have been explored in a series of publications.^97,103^ The Cu-iTMCs containing POP or xantphos and 6,6′-Cl_2_bpy, 6-Brbpy and 6,6′-Br_2_bpy are orange/red emitters in solution and yellow/orange emitters in the solid state. All the halogen-substituted Cu-iTMCs showed longer emission decay lifetimes at 77 K compared to ambient temperatures, consistent with TADF. However, the anticipated red-shift in the emission maximum on going from 298 to 77 K was not observed for [Cu(POP)(6,6′-Cl_2_bpy)][PF_6_], [Cu(xantphos)(6,6′-Cl_2_bpy)][PF_6_] or [Cu(POP)(6,6′-Br_2_bpy)][PF_6_], and this was attributed to the degree of relaxation attained by the emitting T_1_ state in the frozen Me-THF matrix. The emission behaviour of complexes in this series was strongly dependent upon the halogen substitution pattern, and DFT caculations revealed significant effects on the geometry of the emitting triplet state. For this series of Cu-iTMCs, the highest solid-state PLQY values were observed for [Cu(xantphos)(6-Brbpy)][PF_6_], [Cu(POP)(6,6′-Cl_2_bpy)][PF_6_] and [Cu(xantphos)(6,6′-Cl_2_bpy)][PF_6_] (16.3, 14.8 and 17.1%, respectively). Fig. 10a illustrates that one chloro-substituent is accommodated in the ‘bowl’ of the xantphos ligand in [Cu(xantphos)(6,6′-Cl_2_bpy)]^+^, and this is a common structural feature in [Cu(xantphos)(6,6′-R_2_bpy)]^+^ or [Cu(xantphos)(6-Rbpy)]^+^ cations.^100^ Earlier, we noted that effective EL does not necessarily follow from efficient PL. Despite exhibiting solid-state PLQYs in the range 3.9–16.3%, none of the LECs incorporating Cu-iTMCs with 6-Brbpy or 6,6′-Br_2_bpy showed any electroluminescence. In contrast, LECs with [Cu(POP)(6,6′-Cl_2_bpy)][PF_6_] and [Cu(xantphos)(6,6′-Cl_2_bpy)][PF_6_] in the active layers (Fig. 10b) exhibited very short turn-on times (<5 to 12 s) and orange EL (*λ*^em^_max_(EL) = 586 and 587 nm). Values of Lum_max_ of 121 and 259 cd m^−2^ were achieved for [Cu(POP)(6,6′-Cl_2_bpy)][PF_6_] and [Cu(xantphos)(6,6′-Cl_2_bpy)][PF_6_], respectively, with LECs driven using a pulsed current density of 100 A m^−2^.^97*b*^

**Fig. 11:**
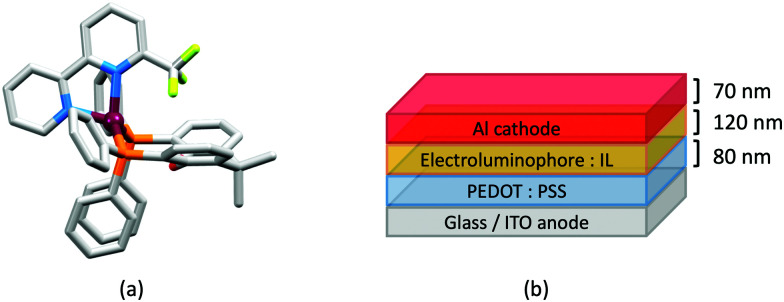
(a) Structure of the [Cu(xantphos)(6-CF_3_bpy)]^+^ cation in the [PF_6_]^−^ salt (CSD refcode VICQUW); the 6-CF_3_ bpy ligand is disordered over two sites and only the major occupancy site is shown. H atoms are omitted. (b) Architecture of LECs containing the Cu-iTMCs with CF_3_-functionalized bpy ligands. IL = [EMIM][PF_6_] and Cu-iTMC: IL molar ratio = 4 : 1. LECs were driven using a pulsed current.

Several Cu-iTMCs containing the CF_3_-functionalized bpy ligands shown in Scheme 4 proved to be very promising, both in terms of PL and EL. Fig. 11a shows the structure of the [Cu(xantphos)(6-CF_3_bpy)]^+^ cation and again we see the hosting of the 6-substituent of the bpy ligand in the bowl-shaped cleft of xantphos, as well as the face-to-face π-stacking of two phenyl rings of different Ph_2_ units of xantphos. As is typical, solid-state PLQYs greatly surpassed solution emission behaviour. The highest PLQY (50.3%) was found for [Cu(xantphos)(4,4′-(CF_3_)_2_-6,6′-Me_2_bpy)][PF_6_]. This compares with only 0.9% for [Cu(xantphos)(4,4′-(CF_3_)_2_bpy)][PF_6_]. However, it compares with 37.3% for [Cu(xantphos)(6,6′-Me_2_bpy)][PF_6_], and once again emphasizes the importance of substituents in the 6,6′-positions of bpy. Both [Cu(POP)(4,4′-(CF_3_)_2_bpy)][PF_6_] and [Cu(xantphos)(4,4′-(CF_3_)_2_bpy)][PF_6_] showed weak emissions, and, in keeping with the trends observed by Weber *et al.*^102^ discussed earlier, it is pertinent to note that the Hammett parameter, *σ*_p_, for CF_3_ is +0.54.^104^ Compounds containing 5,5′-(CF_3_)_2_bpy were poorly emissive, even in the solid state. As well as providing insight into the effects of introducing CF_3_ substituents, our study in 2018^61^ returned to the simple [Cu(POP)(bpy)]^+^ complex first reported in 2011,^59^ and we demonstrated that [Cu(POP)(bpy)][PF_6_] is a TADF emitter. We also looked again at the xantphos-containing compounds [Cu(xantphos)(6-Mebpy)][PF_6_] and [Cu(xantphos)(6,6′-Me_2_bpy)][PF_6_] (first reported in 2016),^37^ and showed that, along with [Cu(xantphos)(bpy)][PF_6_], they also exhibited TADF. LECs (Fig. 11b) containing [Cu(POP)(6-CF_3_bpy)][PF_6_], [Cu(xantphos)(6-CF_3_bpy)][PF_6_] and [Cu(xantphos)(4,4′-(CF_3_)_2_-6,6′-Me_2_bpy)][PF_6_] in their active layers exhibited orange EL (*λ*^em^_max_ in the range 589 to 595 nm). The shortest turn-on time (8 min) was for the LEC with [Cu(xantphos)(4,4′-(CF_3_)_2_-6,6′-Me_2_bpy)][PF_6_] and this also achieved the highest Lum_max_ (131 cd m^−2^).^61^

Jin *et al.* recently compared the PL behaviour of [Cu(POP)(6,6′-(HO)_2_bpy)][ClO_4_] and [Cu(POP)(6,6′-(NC)_2_bpy)][ClO_4_], and also the effects of replacing the wide-bite angle POP by two monodentate phosphanes. Room temperature PLQYs are enhanced on going from two PPh_3_ to POP: for solid samples, PLQY = 10.4% for [Cu(PPh_3_)_2_(6,6′-(HO)_2_bpy)][ClO_4_] *vs.* 16.5% for [Cu(POP)(6,6′-(HO)_2_bpy)][ClO_4_], and 9.2% for [Cu(PPh_3_)_2_(6,6′-(NC)_2_bpy)][ClO_4_] *vs.* 13.5% for [Cu(POP)(6,6′-(NC)_2_bpy)][ClO_4_]. A red-shift in the emission maximum on cooling to 77 K accompanied by extended values of *τ* indicate TADF at room temperature for [Cu(PPh_3_)_2_(6,6′-(HO)_2_bpy)][ClO_4_] and [Cu(PPh_3_)_2_(6,6′-(NC)_2_bpy)][ClO_4_]. The analogous POP complexes also show longer *τ* values at 77 K compared to 298 K. The focus of the study was the ability to tune emission maxima through altering the π-accepting ability of the phosphane ligand and electronic properties of the diimine ligand, and the EL characteristics were not explored.^103^

The bpy-containing derivatives overviewed in this section represent the largest group of N^N ligands in heteroleptic Cu-iTMCs that exhibit TADF and have been tested in LECs. Some of the best-performing LECS have been achieved with this family of electroluminophores, in particular [Cu(xantphos)(4,5,6-Me_3_bpy)][PF_6_], [Cu(xantphos)(6,6′-Me_2_bpy)][PF_6_] and [Cu(xantphos)(4,4′-(CF_3_)_2_-6,6′-Me_2_bpy)][PF_6_]. However, there is often a trade off between fast turn-on of the device and maximum luminance. Fig. 7–11 also reveal significant variability in the layer thicknesses and composition in the LECs; the extent to which these factors affect LEC figures of merit has not been extensively investigated.

### 1,10-Phenanthroline derivatives with POP and xantphos

**Scheme 5:**
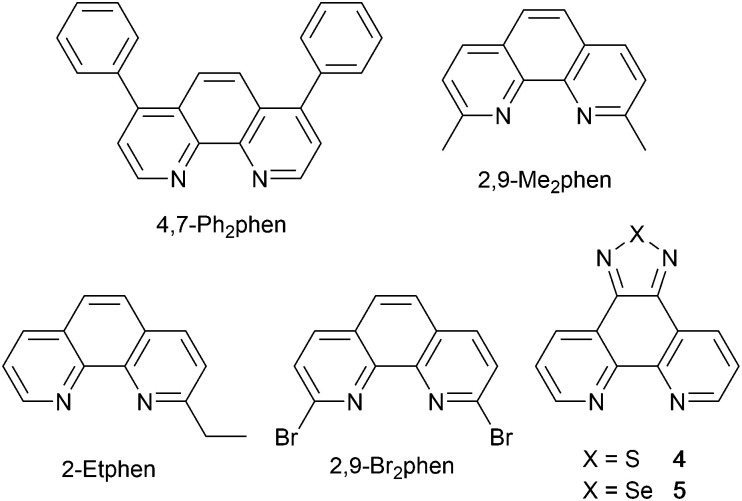
Structures of derivatives of phen used in [Cu(P^P)(N^N)]^+^ complexes, and discussed here.

Although heteroleptic copper(i) complexes incorporating phen and its derivatives are well established (see earlier), investigations of TADF behaviour and investigations of LEC performances appear to be significantly fewer than for bpy-containing Cu-iTMCs. As part of a wider study which provides critical insight into trends in photophysical and electrochemical properties of heteroleptic copper(i) complexes containing phen and 4,7-Ph_2_phen (Scheme 5) including the role of intramolecular π-stacking interactions in improving PLQY, Leoni *et al.* reported the emission spectra of powdered [Cu(POP)(phen)][BF_4_] and of a thin-film between 338 and 78 K. The red-shift in *λ*^em^_exc_(PL) coupled with an increase in the excited state lifetime demonstrate TADF at ambient temperatures. A general point of note is that [Cu(P^P)(N^N)]^+^ complexes containing 4,7-Ph_2_phen tend to show red-shifted emission maxima compared to their phen analogues, *e.g. λ*^em^_exc_(PL) for solid [Cu(POP)(phen)][BF_4_] and [Cu(POP)(4,7-Ph_2_phen)][BF_4_] are 566 and 581 nm, respectively. For this pair of compounds, the introduction of the Ph groups leads to slightly higher PLQY (11.3 *vs.* 15.0%) and longer *τ* (14.08 *vs.* 17.72 μs) in a PMMA thin-film, but has little effect on the powdered material (36.6 *vs.* 35.3%, 12.75 *vs.* 11.72 μs).^60^

**Fig. 12:**
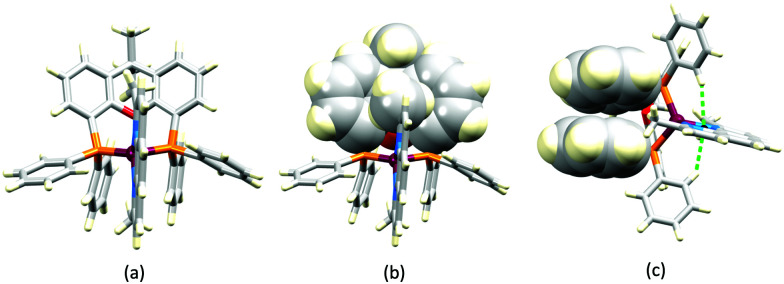
(a) The structure of the [Cu(xantphos)(2,9-Me_2_phen)]^+^ cation in the [BF_4_]^−^ salt (CSD refcode GOZDEH). (b) The same view of the cation as in (a) showing the hosting of one Me substituent in the cavity of the xanthene unit (space-filling representation), and (c) π-stacking interaction between adjacent PPh_2_ units in xantphos (space-filling representation) and CH⋯π contacts (hashed green lines) between Ph units of xantphos and the centroid of the Cu-phen chelate ring.

**Fig. 13:**
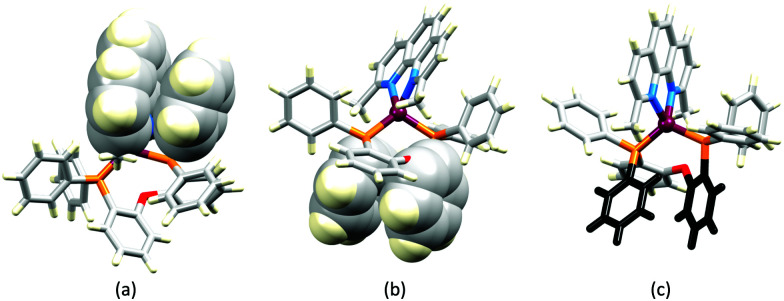
The structures of the [Cu(POP)(2,9-Me_2_phen)]^+^ cation in (a) the tetrakis(3,5-bis(trifluoromethyl)phenyl)borate salt (CSD refcode ARURIP), and (b) [Cu(POP)(2,9-Me_2_phen)][BF_4_] (refcode HOWQAO) with face-to-face π-stacking interaction shown in space-filling representation. (c) The structure of the cation in [Cu(POP)(2,9-Me_2_phen)][BF_4_]·MeCN (CSD refcode CAPLEN); the aromatic rings in black are poorly aligned for an efficient π-stacking contact but nonetheless, the evolution of this interaction is evident.

In order to restrict the flattening of the [Cu(P^P)(phen)]^+^ coordination sphere upon excitation, substituents in the 2- or 2,9-positions of phen are required.^60^ In view of the known relevance of intra-cation π-contacts (see above), it is pertinent to summarize typical structural features of [Cu(P^P)(phen)]^+^ complexes. Fig. 12a displays the structure of the cation in [Cu(xantphos)(2,9-Me_2_phen)][BF_4_]·Et_2_O·CH_2_Cl_2_.^105^ Several features are of note because of their recurrence in other [Cu(xantphos)(phen)]^+^ derivatives. Fig. 12b shows that one substituent of 2,9-Me_2_phen is accommodated in the cavity of the xanthene unit (compare with Fig. 10). In addition, the P^P and N^N ligands associate through CH⋯π contacts (Fig. 12c) between Ph units of xantphos and the {Cu(phen)} unit. In keeping with [Cu(xantphos)(bpy)]^+^ cations (see earlier), [Cu(xantphos)(2,9-Me_2_phen)]^+^ exhibits a π-stacking interaction between adjacent PPh_2_ units within the xantphos domain (Fig. 12c). These interactions should be compared with typical intramolecular interactions in POP-containing derivatives, exemplified by [Cu(POP)(2,9-Me_2_phen)]^+^. The CSD contains the structures of several salts of the latter, and two face-to-face π-stacking contacts recur, but appear to be mutually exclusive (see the later discussion of [Cu(POP)(5)][BF_4_]). The first is between the phen unit and one phenyl ring of a PPh_2_ unit of POP. This interaction occurs in the tetrakis(3,5-bis(trifluoromethyl)phenyl)borate salt of [Cu(POP)(2,9-Me_2_phen)]^+^ (Fig. 13a).^106^ The second involves one PPh_2_ phenyl ring and an arene ring of the POP backbone as seen in [Cu(POP)(2,9-Me_2_phen)][BF_4_] (Fig. 12b, compare with Fig. 6).^107^ In [Cu(POP)(2,9-Me_2_phen)][BF_4_]·MeCN,^108^ the cation (Fig. 12c) shows a π-stacking contact of the type shown in Fig. 12b, but the metrics of the interaction are not optimal. The same is true in [Cu(POP)(2,9-Me_2_phen)][PF_6_]·0.5Et_2_O (CSD refcode CAPZID)^109^ and in [Cu(POP)(2,9-Me_2_phen)][BF_4_]·CH_2_Cl_2_ (CSD refcode EDOCIJ).^110^ These examples illustrate that in crystalline materials, crystallization conditions and the nature of the anion, play important roles in determining, not only lattice packing interactions, but also intra-cation π-contacts. They also indicate that structural data from a single-crystal structure may not necessarily translate to, for example, a thin-film or frozen matrix.

**Fig. 14:**
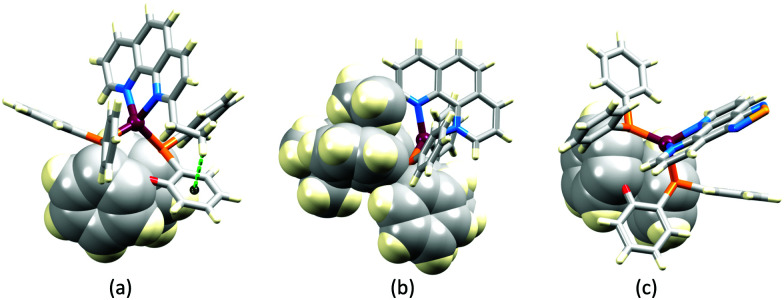
(a) The structure of the [Cu(POP)(2-Etphen)]^+^ cation in the [PF_6_]^−^ salt (CSD refcode PUTSAB) highlighting π-stacking within the POP ligand (space-filling representation) and Et⋯POP CH⋯π contact (hashed green line). (b) The structure of the [Cu(xantphos)(2-Etphen)]^+^ cation in the [PF_6_]^−^ salt (CSD refcode PUTTAC) showing π-stacking within the xantphos ligand (space-filling representation, bottom of diagram) and accommodation of the Et group in the xanthene-bowl (space-filling, left). (c) The structure of the [Cu(POP)(5)]^+^ cation in the [BF_4_]^−^ salt (CSD refcode PUYBET) with the π-stacking within the POP ligand shown in space-filling representation.

The promising PL and EL performances of [Cu(POP)(2)][BF_4_] (2 = 2,9-Bu_2_phen, Scheme 1) were first reported by Armaroli *et al.* in 2006 (see earlier discussion).^58^ Kato and coworkers have reported the emission behaviour of [Cu(xantphos)(2,9-Me_2_phen)][BF_4_]. In CH_2_Cl_2_ solution, the complex has a PLQY of 24% at 298 K, and on going to a PMMA thin-film, *λ*^em^_max_ undergoes a blue-shift (536 to 525 nm) and an increase in PLQY to 29%. At 77 K, *λ*^em^_max_ red-shifts to 541 nm (PMMA film) and *τ* increases from 18.5 to 182 μs (values are from a bi-exponential fit to the decay), consistent with [Cu(xantphos)(2,9-Me_2_phen)]^+^ being a TADF emitter at room temperature.^105^ [Cu(POP)(2-Etphen)][PF_6_] and [Cu(xantphos)(2-Etphen)][PF_6_] (see Scheme 5 for 2-Etphen) also show TADF behaviour. The structure of the [Cu(POP)(2-Etphen)]^+^ cation (Fig. 14a) shows the typical face-to-face π-stacking within the POP ligand, and the ethyl group engages in a C–H⋯π interaction with an arene ring of the POP backbone (Fig. 14a). As in related structures, the bowl-shaped cavity of the xanthene unit in [Cu(xantphos)(2-Etphen)]^+^ cation hosts the substituent on the N^N ligand, and two phenyl rings of xantphos are π-stacked (Fig. 14b). Powdered [Cu(POP)(2-Etphen)][PF_6_] and [Cu(xantphos)(2-Etphen)][PF_6_] emit with *λ*^em^_max_(PL) = 558 and 550 nm, respectively, and with PLQYs of 27.5 and 9.8%. Values of *τ* of 8.7 and 10.2 μs at 298 K increase to 27 and 14 μs, respectively, on cooling to 77 K, but only the emission maximum for [Cu(xantphos)(2-Etphen)][PF_6_] undergoes a red-shift (to 557 nm). The blue-shift observed for [Cu(POP)(2-Etphen)][PF_6_] was attributed to intermolecular matrix effects in the frozen Me-THF glass. The 2-Etphen-containing compounds were incorporated into the active layers of LECs (Table 3) with the same architectures as shown in Fig. 8. The LECs were driven by pulsed current and under an average current density of 100 A m^−2^. The LEC with [Cu(POP)(2-Etphen)][PF6] gave instantaneous EL, with an initial luminance of 273 cd m^−2^ increasing over 25 minutes to a Lum_max_ of 451 cd m^−2^. Although the LEC continued to emit light for 24 hours, the EL decay from Lum_max_ to half this value was rapid (5.7 hours). Compared to the latter, LECs based on [Cu(xantphos)(2-Etphen)][PF_6_] were slower to turn on (122 minutes to reach Lum_max_) and had less intense EL (Lum_max_ = 153 cd m^−2^), but a longer device lifetime (up to 98 hours).^98^ These data should encourage further investigations with [Cu(POP/xantphos)(phen)]^+^-based electroluminophores in which the phen ligand bears a simple alkyl-substitution pattern. The introduction of bromo-substituents leads to weak emitters in solution, but powdered [Cu(POP)(2,9-Br_2_phen)][PF_6_] and [Cu(xantphos)(2,9-Br_2_phen)][PF_6_] (*λ*^em^_max_(PL) = 574 and 554 nm) exhibit PLQYs of 24 and 45%, and excited-state lifetimes of 6.3 and 9.9 μs, respectively. The photophysical properties of the complexes containing 2,9-phen are enhanced with respect to analogues containing 3,8- or 4,7-dibromo-1,10-phenanthrolines.^111^ More detailed investigations of these compounds have not been carried out.

In [Cu(PPh_3_)_2_(4)][BF_4_], [Cu(POP)(4)][BF_4_], [Cu(PPh_3_)_2_(5)][BF_4_] and [Cu(POP)(5)][BF_4_], ligands 4 and 5 (Scheme 5) lack the steric hindrance imposed by 2,9-substituents in phen, and Farias *et al.* have assessed the effects of the chalcogen atoms in 4 and 5 on the photophysical properties of the complexes. The solid-state structure of [Cu(POP)(5)]^+^ shows the expected π-stacking of phenyl and arene rings in POP, and Fig. 14c also reveals the close approach of one PPh_2_ phenyl ring to the phen domain. However, no π-stacking interaction is established. In [Cu(POP)(4)][BF_4_] (CSD refcode PUXZUG), the dominant intra-cation packing is again within the POP framework. Compared to [Cu(PPh_3_)_2_(phen)][BF_4_] and [Cu(POP)(phen)][BF_4_], complexes containing 4 and 5 exhibit lower PLQYs (4 and 8% *vs.* 11% for the PPh_3_ derivatives, and 6 and 8% *vs.* 13% for the POP derivatives, all in PMMA films), and excited-state lifetimes are shorter. Using steady-state and time-resolved spectroscopies, Farias *et al.* confirmed that the compounds with 4 and 5 are TADF emitters, and showed that the incorporation of the Se atom contributed to decreasing the PL lifetime to *ca.* 800 ns. This was claimed to be the lowest reported to date (2020) among similar TADF materials.^112^

### Pyrazolyl pyridine derivatives with POP

Typically, the bpy and phen-containing compounds described in the previous sections are orange, yellow or green emitters. Moving from bpy or phen to an N^N ligand comprising pyridine connected to a 5-membered N-heterocycle alters the bite angle of the chelating ligand and increases its ligand-field strength. This is a proven means of shifting emissions of [Cu(P^P)(N^N)]^+^ complexes towards the blue.

**Scheme 6:**
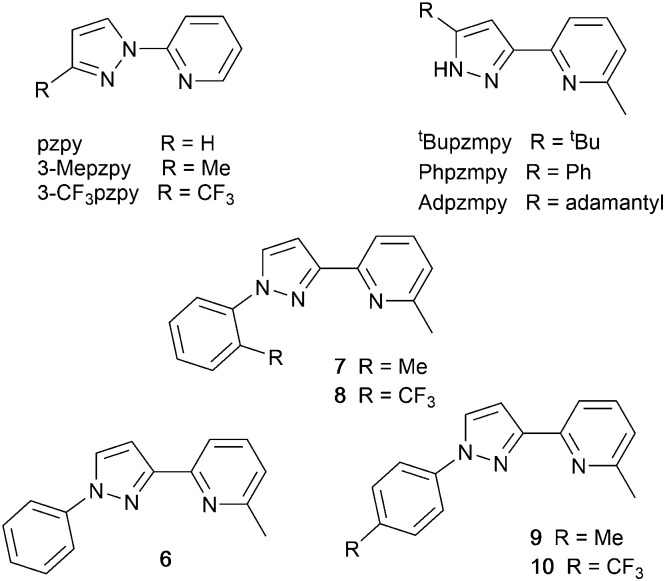
Structures of pyrazolyl pyridine derivatives used in [Cu(P^P)(N^N)]^+^ complexes.

**Fig. 15:**
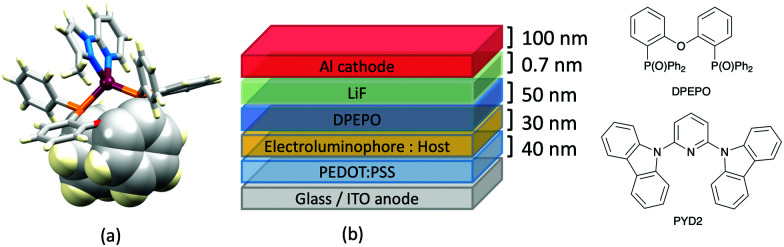
(a) Structure of the complex cation in [Cu(POP)(3-Mepzpy)][BF_4_]·0.5CH_2_Cl_2_ (CSD refcode VIZKEW) with Ph⋯arene π-stacking within the POP ligand shown in space-filling representation. (b) Architecture of solution-processed OLEDs with [Cu(POP)(pzpy)][BF_4_] derivatives in the active layer. DPEPO = hole-blocking layer; active layer = 20 wt% Cu-iTMC in either DPEPO or PYD2 as host.

**Fig. 16:**
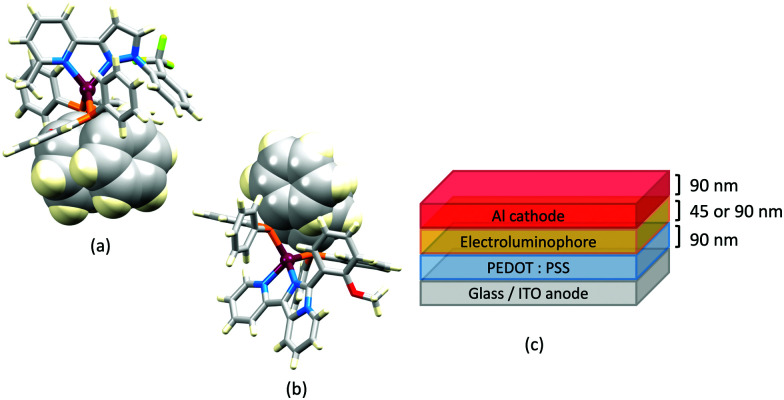
Structures of the complex cations in (a) [Cu(POP)(8)][BF_4_]·EtOH (CSD refcode JUFNOP) and (b) [Cu(POP)(11)][PF_6_] showing Ph⋯arene π-stacking in the POP ligand (space-filling representation). (c) LEC architecture for testing with [Cu(POP)(11)][PF_6_] in the active layer; LECs were driven with a pulsed current.

Series of strongly green/blue- or blue-emitting [Cu(POP)(N^N)][BF_4_] compounds have been reported in which N^N is pzpy, 3-Mepzpy or 3-CF_3_-pzpy (Scheme 6 and Fig. 15a)^113^ and ^*t*^Bupzmpy, Phpzmpy or Adpzmpy (Scheme 6).^114^ For the first series with pzpy, 3-Mepzpy or 3-CF_3_-pzpy, PLQYs of up to 45% were observed in deaerated CH_2_Cl_2_ solution, and for solid-state [Cu(POP)(N^N)][BF_4_] (*λ*^em^_max_ = 490, 465 and 492 nm for N^N = pzpy, 3-Mepzpy and 3-CF_3_-pzpy, respectively), the PLQYs were 56, 87 and 75%, respectively. The temperature dependence of the emission lifetimes and red-shifts in *λ*^em^_max_(PL) on going from 298 to 77 K, established TADF at ambient temperatures with Δ*E*_ST_ lying in the range 0.17–0.18 eV. The highest solid-state PLQY (87% for N^N = 3-Mepzpy) corresponded to the shortest *τ* value (12.2 μs). Solution-processed OLEDs were fabricated using the three [Cu(POP)(N^N)][BF_4_] salts with either DPEPO or PYD2 (also abbreviated in the literature to 26mCPy) as host materials (Fig. 15b). The best EL performance was found for [Cu(POP)(3-CF_3_-pzpy)][BF_4_]: PYD2 with the relative LUMO energies of [Cu(POP)(3-CF_3_-pzpy)][BF_4_] (−2.49 eV) and PYD2 (−2.2 eV) contributing to an efficient electron–hole recombination pathway. A Lum_max_ of 2033 cd m^−2^ was achieved for this device.^113^ This work was extended to [Cu(POP)(N^N)][BF_4_] with N^N = 6–10 (Scheme 6) and these are highly-efficient TADF emitters. Structural characterization of all five compounds confirms that the steric requirements of the substituents in the pzpy ligands do not preclude intra-POP π-stacking (Fig. 16a). Solid materials were intense blue-green or blue emitters (*λ*^em^_max_ in the range 464 to 481 nm at 298 K, PLQY = 82–99%) with a red-shift for spectra recorded at 77 K (*λ*^em^_max_ in the range 487 to 513 nm). On doping in PMMA, all the Cu-iTMCs show sky-blue emissions, and for all but [Cu(POP)(8)][BF_4_], *τ* values increase with decreasing rigidity of the matrix.^115^

### Imidazolyl pyridine derivatives with POP and xantphos

**Scheme 7:**
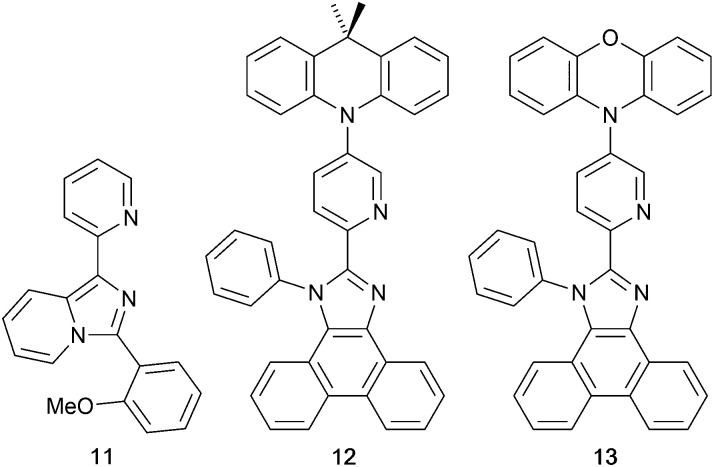
Structures of imidazolyl pyridine derivatives used in [Cu(P^P)(N^N)]^+^ complexes.

The search for blue-emitting Cu-based LECs takes us from pyrazolyl- to imidazolyl-containing Cu-iTMCs. Ligand 11 (Scheme 7) was incorporated into [Cu(POP)(11)][PF_6_] which gave a deep-blue emission at *ca.* 450 nm in solution, thin-film and the solid state. The structure of the [Cu(POP)(11)]^+^ cation showed typical features (Fig. 16b). Unexpectedly, LECs fabricated with [Cu(POP)(11)][PF_6_] in the active layer (Fig. 16c) were yellow emitters (EL = 550 nm), with luminances which depended both on the thickness of the active layer and on the pulsed current (1, 2.5, 5 or 7.5 mA). Detailed studies concluded that the origin of this large PL-to-EL shift lay in the fact that [Cu(POP)(11)]^+^ did not exhibit TADF because of the exclusively ligand-centred character of the excited states. The lack of any charge-transfer character in the excited states resulted, respectively, in a blue-fluorescent and yellow phosphorescent PL and EL.^116^ Ligands 12 and 13 were designed to possess intra-ligand charge-transfer character. Each of [Cu(POP)(12)][BF_4_], [Cu(POP)(13)][BF_4_], [Cu(xantphos)(12)][BF_4_] and [Cu(xantphos)(13)][BF_4_] exhibits TADF. In solution at room temperature, values of *λ*^em^_max_ lie between 514 to 537 nm (*λ*_exc_ = 365 nm) with an N^N ligand-dominated excited state. Given the steric hindrance of the ligands which militate against significant flattening of the Cu(i) coordination sphere, the low PLQYs (4.2–9.5%) were explained in terms of torsional dynamics of the N^N ligand framework in solution. PLQYs (42–71%) and decay lifetimes were significantly enhanced on going to thin films. Emission data for [Cu(xantphos)(13)][BF_4_] were recorded from 77 K to ambient temperature and confirmed TADF with longer *τ* values at lower temperatures; the value of Δ*E*_ST_ = 0.04 eV (*ca.* 300 cm^−1^) is also consistent with TADF. Solution-processed, multilayer OLEDs were fabricated with [Cu(xantphos)(12)][BF_4_] or [Cu(POP)(13)][BF_4_] hosted in bis(9*H*-carbazol-9-yl)pyridine in the active layer and using different doping levels. The maximum external quantum efficiency (EQE = 7.96%) was achieved with [Cu(POP)(13)][BF_4_].^9^

### Tri- and tetrazolyl pyridine derivatives with POP, xantphos and PPh_3_

**Scheme 8:**
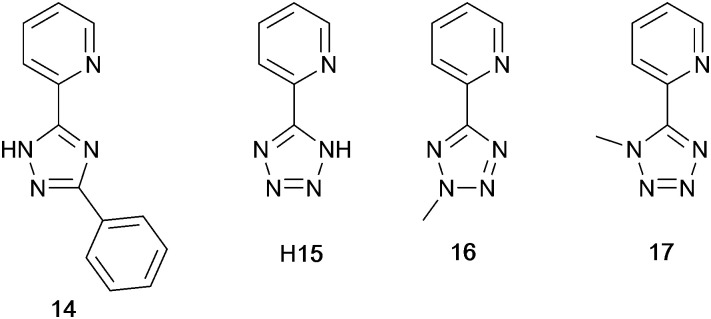
Structures of tri- and tetrazolyl pyridine derivatives used in [Cu(P^P)(N^N)]^+^ complexes.

The families of N^N ligands with pyridine connected to a 5-membered N-heterocycle include a number of triazole and tetrazole derivatives (Scheme 8), and, of course, the presence of an NH unit gives the potential for deprotonation accompanying coordination and the formation of a neutral rather than cationic Cu(i) heteroleptic complexes. The p*K*_a_ values of triazole (p*K*_a_ = 9.4) and tetrazole (p*K*_a_ = 4.9) account for the fact that in the examples below, H15 binds to Cu(i) as the conjugate base while 14 remains protonated.

Xu *et al.* have reported the TADF emitters [Cu(PPh_3_)_2_(14)][BF_4_] and [Cu(POP)(14)][BF_4_]. The low intensity absorption maxima at 362 and 342 nm, respectively, were assigned to MLCT and ligand-to-ligand (LLCT) transitions. Solution emissions were weak, but in the solid state, emissions with *λ*^em^_max_(PL) = 490 and 512 nm with PLQYs of 89.97 and 27.82%, respectively, for [Cu(PPh_3_)_2_(14)][BF_4_] and [Cu(POP)(14)][BF_4_] were observed. Interestingly, it is the PPh_3_, and not the wide bite-angle POP, derivative that performs the better of the two compounds. At 298 K, the excited-state decay times are 23.6 and 13.0 μs and these increase to 269.0 and 210.1 μs at 77 K. Values of Δ*E*_ST_ are 0.09 and 0.04 eV (*ca.* 700 and 300 cm^−1^), and these data, accompanied by the red-shift in *λ*^em^_max_(PL) to 508 and 520 nm for [Cu(PPh_3_)_2_(14)][BF_4_] and [Cu(POP)(14)][BF_4_], respectively, support TADF at ambient temperatures. Multilayer OLEDs were processed using these ionic complexes with 4,4′,4′′-tris(*N*-carbazolyl)triphenylamine as the host material. The OLEDs with [Cu(PPh_3_)_2_(14)][BF_4_] and [Cu(POP)(14)][BF_4_] showed green emissions (*λ*^em^_max_(EL) to 520 and 539 nm), and the POP-containing electroluminophore yielded the higher Lum_max_ (1871 *vs.* 1437 cd m^−2^), with the smaller Δ*E*_ST_ being a contributing factor.^10^ A small Δ*E*_ST_ (*ca.* 0.18 eV, 1500 cm^−1^) separation is also found for the neutral compound [Cu(POP)(15)], and a detailed theoretical investigation demonstrated that low-frequency vibrational modes associated with the torsional motion of the POP and N^N ligands lead to substantial Huang–Rhys factors^117^ and, thereby, to rapid ISC and RISC (see Fig. 2).^118^ Related studies which address the effects of both intramolecular and intermolecular interactions on photophysical properties, have been carried out on the cationic TADF emitters [Cu(POP)(16)]^+^ and [Cu(POP)(17)]^+^.^119^ These complexes exhibit both aggregation induced emission (AIE) and TADF. For the former phenomenon, enhanced emission in the solid state (as opposed to in solution) arises from molecular aggregation that restricts intramolecular rotation. [Cu(POP)(16)][BF_4_] and [Cu(POP)(17)][BF_4_] are virtually non-emissive in CH_2_Cl_2_ solutions, but thin-films spin-coated from CH_2_Cl_2_ solution were bright emitters (*λ*^em^_max_(PL) = 533 and 572 nm for N^N = 16 and 17) with very short decay times. In this form, the Cu-iTMCs were principally TADF emitters over a 320–170 K temperature range. Between 170 and 80 K, phosphorescence was the dominant decay path. A comparison of the PL of thin-films made with [Cu(POP)(16)][BF_4_] and [Cu(POP)(17)][BF_4_] in low- or high-molecular weight PMMA showed that the different PMMA hosts were able to suppress molecular vibrations to different extents. In low molecular weight PMMA, TADF contributed little to the emission, with vibrational quenching being most effective and ambient (rather than lower) temperatures. Moving to the high molecular weight PMMA leads to greater suppression of molecular vibrations within the Cu-iTMCs and opens up the TADF pathway. Ligands 16 and 17 differ only in the position of the methyl substituent in the tetrazole (Scheme 8). This leads to significant differences in crystal packing with the inter-cation interactions resulting in a 3D-supramolecular assembly in [Cu(POP)(16)][BF_4_], but to 1D-chains in [Cu(POP)(17)][BF_4_]. It follows that there is less distortion of the excited state of the complex with 16, and consistent with this notion is the fact that the solid-state PLQY of [Cu(POP)(16)][BF_4_] (47.1%) is higher than that of [Cu(POP)(17)][BF_4_] (9.4%). Both complexes are put forward as potential candidates for lighting devices.^120^

### Derivatives of di(pyridin-2-yl)sulfane and related N^N ligands with POP

**Scheme 9:**
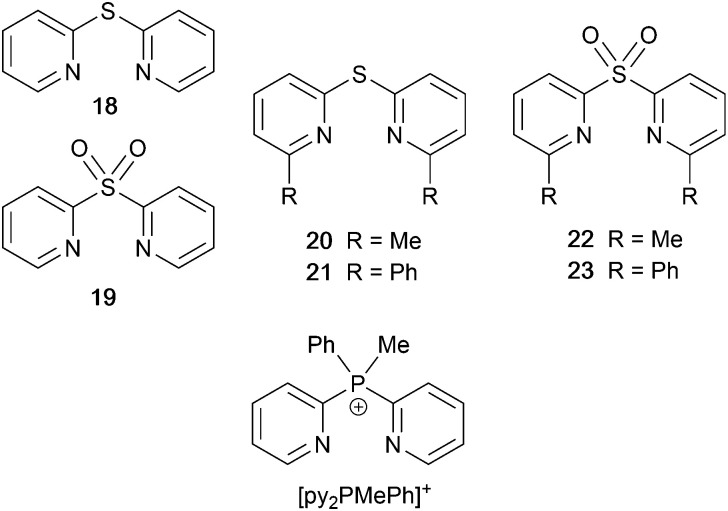
Structures of di(pyridin-2-yl)sulfane (18) and di(pyridin-2-yl)sulfone (19) and some functionalized derivatives, and the structure of [py_2_PMePh]^+^. See also Scheme 16.

**Fig. 17:**
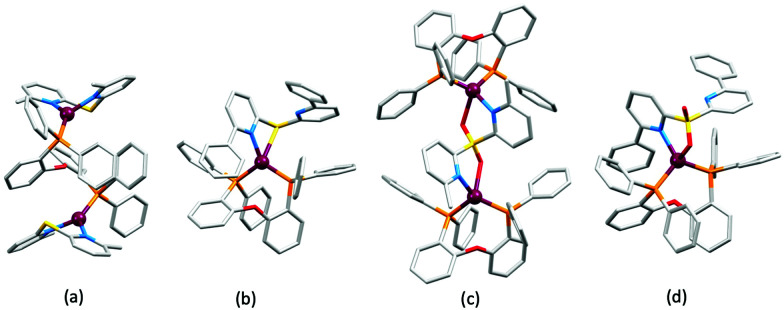
Structures of the complex cations (a) [Cu_2_(20)_2_(μ-POP)]^2+^ (CSD refcode SONBIJ), (b) [Cu(POP)(21)]^+^ (refcode SONBOP), (c) [Cu_2_(POP)_2_(μ-22)]^2+^ (refcode SONBAB), and (d) [Cu(POP)(23)]^+^ (refcode SONBUV). Each was structurally characterized as the [BF_4_]^−^ salt, and in the figures, H atoms are omitted for clarity.

Scheme 9 shows a series of new N^N ligands incorporated into heteroleptic copper(i) coordination compounds, and designed with S in two different oxidation states. Unexpectedly, whereas 18 and 19 behave as N,N′-chelating ligands and form distorted tetrahedral [Cu(POP)(N^N)]^+^ complexes, ligand 20 gives a dinuclear complex with three-coordinate Cu(i) and bridging POP (Fig. 17a), while 21 behaves as an N,S-chelating ligand (Fig. 17b), and 22 and 23 bind through N- and O-donors in mono- and dinuclear complexes, respectively (Fig. 17c and d). All the compounds (as [BF_4_]^−^ salts) are weakly emissive in CH_2_Cl_2_ solution. Of the solution emissions, the most blue-shifted is for [Cu(POP)(21)][BF_4_] (*λ*^em^_max_(PL) = 456 nm) and this emission band is the only one to show structure; radiative decay from a ligand-centred excited state was proposed. Thin-films drop-cast from MeOH solutions of the complexes produced more intense emissions than in solution, with values of *λ*^em^_max_ in the range 518–572 nm. For each pair of complexes with sulfane and sulfone ligands, the emission undergoes a red-shift on going from S to SO_2_ unit. The highest solid-state PLQYs are for [Cu_2_(POP)_2_(μ-22)][BF_4_]_2_ (14%) and [Cu(POP)(23)][BF_4_] (20%). With the exception of [Cu(POP)(21)][BF_4_], the emission maxima are red-shifted on going from 298 to 77 K, and PL lifetimes increase, consistent with TADF; in all cases, Δ*E*_ST_ < 0.2 eV (<1600 cm^−1^). For [Cu(POP)(21)][BF_4_], *λ*^em^_max_(PL) = 526 nm at 298 K and 528 nm at 77 K, while *τ* decreases upon cooling. As with the solution emission (see above), the data point to a ligand-centred emission. While not tested in LECs, the TADF emitters are promising candidates for such application, and also open up the possiblity of using sulfone-based N,O-coordinated diimine ligands.^121^

Use of the N^N ligands 18 and 19 has been extended by Gaillard, Costa and coworkers to include analogous ligands with bridging CMe_2_, NH, O and PPh units. This series of [Cu(POP)(N^N)][PF_6_] complexes along with [Cu(POP)(py_2_PMePh)][PF_6_]_2_ (see Scheme 9) are weak emitters in solution, and for N^N = 18 or 19, powdered samples also showed PLQY values <1%. However, for the remaining compounds, solid-state PLQYs were in the range 17–60%, with the maximum (also in thin film) being for the copper(i) complex with N^N = py_2_O. With calculated values of Δ*E*_ST_ in the range 0.05–0.22 eV (*ca.* 400–1700 cm^−1^), the [Cu(POP)(N^N)]^+^ and [Cu(POP)(py_2_PMePh)]^2+^ complexes were expected to exhibit TADF, and this was confirmed experimentally for representative examples. [Cu(POP)(py_2_PMePh)][PF_6_]_2_ is singled out from the series as exhibiting good electrochemical stability and high ionic conductivity, and a red-shifted emission (*λ*^em^_max_(PL) = 606 nm in the solid state). LECs with [Cu(POP)(py_2_PMePh)][PF_6_]_2_ as the luminophore showed a yellow emission with a Lum_max_ of *ca.* 60 cd m^−2^ and an efficacy of 0.2 cd A^−1^.^122^

### Complexes with wide-bite angle bis(phosphanes) other than POP and xantphos

**Scheme 10:**
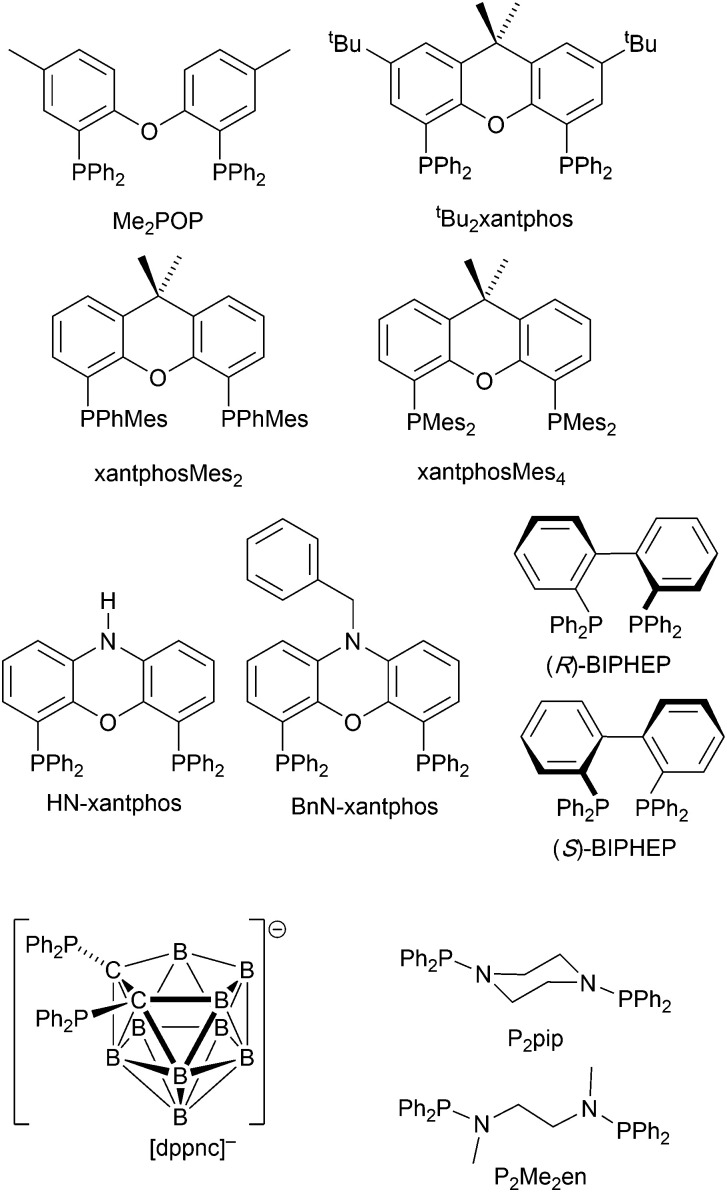
Structures of wide-bite angle bis(phosphanes) used in [Cu(P^P)(N^N)]^+^ complexes. See Scheme 1 for POP and xantphos.

The commercial accessibility of POP and xantphos contribute towards their being the most popular wide-bite angle bis(phosphanes) in heteroleptic copper(i) compounds. However, the pool of ligands that fall in this category is large,^56^ and in this section, we focus on other sterically demanding P^P ligands (Scheme 10) that have been used to stablize [Cu(P^P)(N^N)]^+^ complexes.

**Fig. 18:**
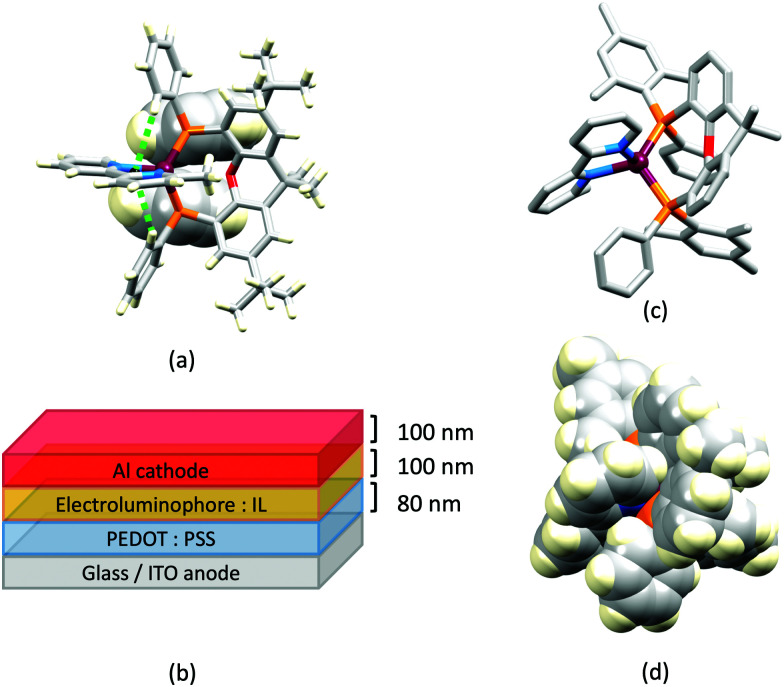
(a) Structure of the cation in [Cu(^*t*^Bu_2_xantphos)(6-Mebpy)][PF_6_] (CSD refcode HIJRAW). π-Stacking between phenyl rings of different PPh_2_ groups (space-filling representation) and C–H_phenyl_⋯bpy interactions (green hashed lines) are highlighted. (b) Architecture of the LECs containing [Cu(^*t*^Bu_2_xantphos)(N^N)][PF_6_] and [Cu(xantphosMes_2_)(N^N)][PF_6_]; IL = [EMIM][PF_6_], and Cu-iTMC: IL molar ratio = 4 : 1; LECs were driven using a pulsed current. (c) Structure of the cation in [Cu(xantphosMes_2_)(6-Mebpy)][PF_6_] (refcode YITSOM); H atoms omitted, and (d) a space-filling representation of the [Cu(xantphosMes_2_)(6-Mebpy)]^+^ cation in the same orientation as in (c).

Earlier, we described the small Δ*E*_ST_ (*ca.* 0.18 eV, 1500 cm^−1^) separation in the tetrazole-containing [Cu(POP)(15)], and theoretical studies indicate that introducing methyl substituents into the backbone of POP to give Me_2_POP (Scheme 15) has little effect on the T_1_–S_1_ energy gap.^118^ There appear to be no experimental investigations of the effects on the properties of [Cu(POP)(N^N)]^+^-type complexes of modifying the POP ligand, and there are only limited studies on complexes incorporating modified xantphos ligands. In 2019, we reported the use of the ^*t*^Bu_2_xantphos ligand (Scheme 15). The strategy behind introducing *tert*-butyl groups was to produce greater spatial separation of Cu-iTMC cations in the active layer in a LEC. Across the series [Cu(^*t*^Bu_2_xantphos)(bpy)][PF_6_], [Cu(^*t*^Bu_2_xantphos)(6-Mebpy)][PF_6_] (Fig. 18a) and [Cu(^*t*^Bu_2_xantphos)(6,6′-Me_2_bpy)][PF_6_], the Cu^+^ oxidation moves to higher potential (+0.76, +0.83, +0.85 V *vs.* Fc/Fc^+^) in keeping with the increased steric demands of the bpy ligand. Both the solution and solid-state emission maxima for [Cu(^*t*^Bu_2_xantphos)(N^N)][PF_6_] are blue-shifted on going from bpy to 6-Mebpy to 6,6-Me_2_bpy: in solution, *λ*^em^_max_(PL) = 652, 605, 566 nm, and for powder, *λ*^em^_max_(PL) = 584, 552, 522 nm. Emission spectra at 77 K exhibit maxima at 597, 578 and 555 nm, all red-shifted with respect to the solids at 298 K. This, and the extended *τ* values on going from 298 to 77 K (1.95 to 27.6 μs for N^N = bpy, 6.32 to 56.3 μs for 6-Mebpy, 13.8 to 92.1 μs for 6,6′-Me_2_bpy) are consistent with TADF behaviour. Both solid-state and frozen matrix emission decays were fitted biexponentially. The room temperature solid-state PLQYs for these [Cu(^*t*^Bu_2_xantphos)(N^N)][PF_6_] compounds range from 3 to 59%, although in thin-films, values are lower. The trend of increasing PLQY with increasing steric demands of the N^N ligand are replicated in the luminances of LECs fabricated as shown in Fig. 18b. The LECs had a fast turn-on times (1–4.5 minutes) to reach Lum_max_ of 20, 230 and 370 cd m^−2^ for N^N = bpy, 6-Mebpy and 6,6′-Me_2_bpy, respectively. However, the low EQE of 1.0% for the brightest LECs indicates that non-radiative losses dominate in the recombination of injected electrons and holes, and a comparison of the performances of LECs containing [Cu(^*t*^Bu_2_xantphos)(6,6′-Me_2_bpy)][PF_6_] and [Cu(xantphos)(6,6′-Me_2_bpy)][PF_6_] reveals with the ^*t*^Bu groups have a negligible influence.^123^

Modification of the xantphos ligand has also involved replacing the PPh_2_ units by PMesPh and PMes_2_ (Mes = mesityl). The wide-bite angle ligand xantphosMes_4_ (Scheme 10) proved to be too sterically demanding to form [Cu(xantphosMes_4_)(N^N)]^+^ even for unsubstituted bpy. For xantphosMes_2_ (Scheme 10), it was possible to isolate [Cu(xantphosMes_2_)(N^N)][PF_6_] with N^N = bpy and 6-Mebpy (Fig. 18c), but not 6,6′-Me_2_bpy. The combined steric demands of the xantphosMes_2_ and 6-Mebpy ligands can be appreciated by looking at Fig. 18d. Comparisons of PL data for [Cu(xantphosMes_2_)(bpy)][PF_6_] and [Cu(xantphosMes_2_)(6-Mebpy)][PF_6_] at 298 K (powder) and 77 K (frozen matrix) confirmed red-shfiting of emission maxima (589 to 594 nm for bpy, 547 to 587 nm for 6-mebpy) and increased decay times (1.19 to 20.0 μs for bpy, 6.62 to 19.7 μs for 6-Mebpy), indicative of TADF behaviour at ambient temperatures. LECs with the architecture shown in Fig. 18b were tested with [Cu(xantphosMes_2_)(bpy)][PF_6_] and [Cu(xantphosMes_2_)(6-Mebpy)][PF_6_] in the active layer, but no EL was observed for the former device. This correlates with the low PLQY (1.9%) of solid [Cu(xantphosMes_2_)(bpy)][PF_6_] at room temperature. The LEC with [Cu(xantphosMes_2_)(6-Mebpy)][PF_6_] exhibited a fast turn-on time (it reached Lum_max_ in <1 minute) but only a moderate Lum_max_ (50 cd m^−2^).^123^ Thus, the modifications of xantphos, either with bulky peripheral groups, or with P-substituents more sterically demanding that phenyl, do not appear to enhance PL or EL properties, and these latter investiagtions tend to suggest that, with xantphos at least, the choice of N^N ligand is the dominant factor.

**Fig. 19:**
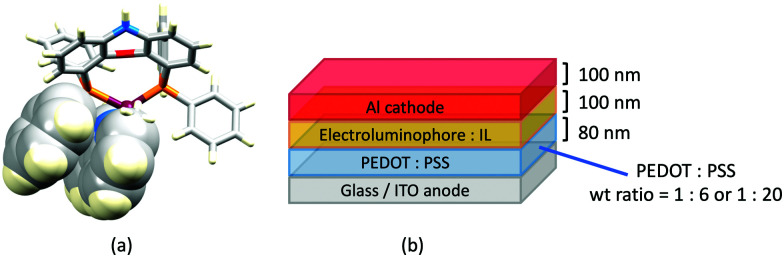
(a) Structure of the cation in [Cu(HN-xantphos)(6-Mebpy)][PF_6_] with the Ph⋯bpy π-stacking interaction shown in space-filling representation (CSD refcode TUHXEC). (b) Architecture of the LECs containing [Cu(BnN-xantphos)(6,6′-Me_2_bpy)][PF_6_]; IL = [EMIM][PF_6_], and Cu-iTMC: IL molar ratio = 4 : 1; two compositions of the hole-injection layer were used; LECs were driven using a pulsed current.

Despite being commercially available, HN-xantphos (Scheme 10) has received far less attention than xantphos, especially with respect to heteroleptic copper(i) coordination compounds.^124–129^ We focus on those studies directed towards applications in LECs. We have already seen that among some of the best performing LEC-emitters are [Cu(xantphos)(6-Mebpy)][PF_6_] and [Cu(xantphos)(6,6′-Me_2_bpy)][PF_6_], and we reported a comparison of the PL and EL behaviours of their HN-xantphos analogues in 2020, along with the effects of replacing the NH by an *N*-benzyl group (BnN-xantphos, Scheme 10). The crystal structure of [Cu(HN-xantphos)(6-Mebpy)][PF_6_] reveals an inter-ligand face-to-face π-stacking interaction between a phenyl ring of a PPh_2_ unit and the bpy domain (Fig. 19a). A similar intramolecular interaction occurs in [Cu(BnN-xantphos)(6,6′-Me_2_bpy)][PF_6_]. In solution, [Cu(BnN-xantphos)(N^N)][PF_6_] with N^N = bpy, 6-Mebpy and 6,6′-Me_2_bpy are weakly emissive; the analogous compounds containing HN-xantphos are unstable in CH_2_Cl_2_ with respect to ligand redistribution. The emission data for powdered samples are given in Table 4 and show similar trends to those for [Cu(xantphos)(N^N)][PF_6_] with N^N = bpy, 6-Mebpy and 6,6′-Me_2_bpy in Table 1 with *λ*^em^_max_(PL) undergoing a blue shift, and PLQY and *τ* increasing on going from bpy to 6-Mebpy to 6,6′-Me_2_bpy. Although the calculated values of Δ*E*_ST_ (0.14–0.20 eV, *ca.* 1100–1600 cm^−1^) are small enough to allow RISC to occur (Fig. 2), an analysis of the oscillator strengths for electronic transitions suggests that the RISC process leads to non-radiative decay rather than TADF. Because of the high solid-state PLQY of [Cu(BnN-xantphos)(6,6′Me_2_bpy)][PF_6_], this compound was selected for inclusion in the active layer of LECs, the latter being fabriacted as shown in Fig. 19b. An interesting aspect of this investigation was the effect of using commercial PEDOT: PSS with different weight ratios in the hole-injection layer. The time to reach a luminance of 100 cd m^−2^ was only 7 s with 1 : 20 PEDOT: PSS for LECs driven using current densities of 50 or 100 A m^−2^. In contrast, this turn-on time was 185 s (at 50 A m^−2^) or 12 s (at 100 A m^−2^) for the 1 : 6 PEDOT: PSS. It is the latter composition that is most commonly employed in LECs. Values of Lum_max_ also depended on the PEDOT: PSS composition, reaching 203 or 355 cd m^−2^. Overall, the LECs with [Cu(BnN-xantphos)(6,6′-Me_2_bpy)][PF_6_] showed good lifetimes, intense EL and EQE >1%, making them some of the best performing devices with [Cu(P^P)(N^N)]^+^ emitters.^127^

**Table 4:** Room temperature PL emission maxima, PLQY values and decay lifetimes (*τ*) for solid-state [Cu(RN-xantphos)(Me_*n*_bpy)]^+^ complexes (R = H, Bn; *n* = 0, 1, 2). Data from Arnosti *et al.*^127^

Compound	*λ* ^em^_max_/nm^a^	PLQY/%	*τ*/μs
[Cu(HN-xantphos)(bpy)][PF_6_]	555	5	1.77
[Cu(HN-xantphos)(6-Mebpy)][PF_6_]	535	17	10.8
[Cu(HN-xantphos)(6,6′-Me_2_bpy)][PF_6_]	518	14	14.2
[Cu(BnN-xantphos)(bpy)][PF_6_]	575	2	1.8
[Cu(BnN-xantphos)(6-Mebpy)][PF_6_]	550	8	7.5
[Cu(BnN-xantphos)(6,6′-Me_2_bpy)][PF_6_]	520	55	17.4

a *λ*_exc_ = 365 nm.

**Fig. 20:**
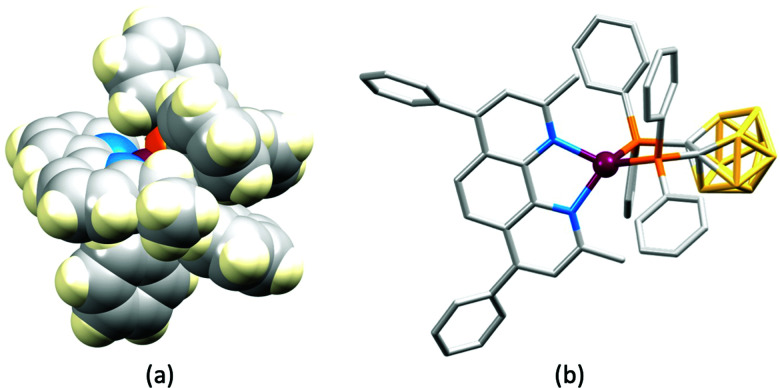
(a) Structure of the cation in [Cu(BIPHEP)(6-Etbpy)][PF_6_] in space-filling representation (CSD refcode WOXHEZ). (b) Structure of [Cu(dppnc)(2,9-Me_2_-4,7-Ph_2_phen)] (CSD refcode MUJXOG) with H atoms omitted.

The chiral BIPHEP ligand (Scheme 10) is also commercially available and the racemic form was used to prepare [Cu(BIPHEP)(N^N)][PF_6_] in which N^N = bpy, 6-Mebpy, 6-Etbpy and 5,5′-Me_2_bpy. As we have seen, [Cu(P^P)(6,6′-Me_2_bpy)][PF_6_] compounds are some of the most emissive of this family, but attempts to synthesize [Cu(BIPHEP)(6,6′-Me_2_bpy)][PF_6_] gave only inseparable mixtures of homo- and heteroleptic complexes, presumably due to excessive steric demands of the two ligands within the four-coordinate environment of copper(i). Although in the solid state, the steric demands of the ligands in the [Cu(BIPHEP)(6-Etbpy)]^+^ cation protect the Cu(i) centre, there are no face-to-face π-stacking interactions (Fig. 20a). In contrast, in [Cu(BIPHEP)(bpy)]^+^, [Cu(BIPHEP)(6-Mebpy)]^+^ and [Cu(BIPHEP)(5,5′-Me_2_bpy)]^+^, one PPh_2_ phenyl ring engages in a stacking contact with one ring of the BIPHEP backbone. As is typical of most [Cu(P^P)(N^N)][X] salts, solution emissions of the BIPHEP derivatives were very weak, but in the solid state, PLQYs are 3–14%, the highest being for [Cu(BIPHEP)(5,5′-Me_2_bpy)][PF_6_]. The yellow emitters have emission maxima at 566 nm for N^N = bpy, 568 nm for 6-Mebpy, 582 for 6-Etbpy, and 558 nm for 5,5′-Me_2_bpy. On going from 298 to 77 K, these shift to 615, 595, 590 and 600 nm, respectively, and the decay lifetimes increase from 3 to 45 μs for [Cu(BIPHEP)(6-Mebpy)][PF_6_], 1 to 53 μs for [Cu(BIPHEP)(6-Etbpy)][PF_6_], and 8 to 49 μs for [Cu(BIPHEP)(5,5′-Me_2_bpy)][PF_6_], consistent with TADF at ambient temperatures. Despite the large increase in *τ* for [Cu(BIPHEP)(6-Etbpy)][PF_6_], this complex showed only a small red-shift in *λ*^em^_max_ (583 to 590 nm) on going from 298 to 77 K, suggesting that, for example, non-radiative decay is operative. For N^N = bpy, despite the red-shift in *λ*^em^_max_(PL) from 566 to 615 nm (298 to 77 K), there was little change in the decay lifetime (3 to 7 μs). With the highest PLQY of the series, [Cu(BIPHEP)(5,5′-Me_2_bpy)][PF_6_] was used as the electroluminophore in a LEC of configuration glass-ITO/PEDOT:PSS/Cu-iTMC/Al but the highest Lum_max_, even with a current density of 100 A m^−2^, was only 12 cd m^−2^ and the maximum EQE was 0.03%.^130^ Since bpy, 6-Mebpy, 6-Etbpy and 5,5′-Me_2_bpy have proven promising with POP, xantphos and BnN-xantphos, it might be concluded that BIPHEP is not a high priority wide-bite angle ligand for further exploration in these types of luminescent compounds.

**Scheme 11:**
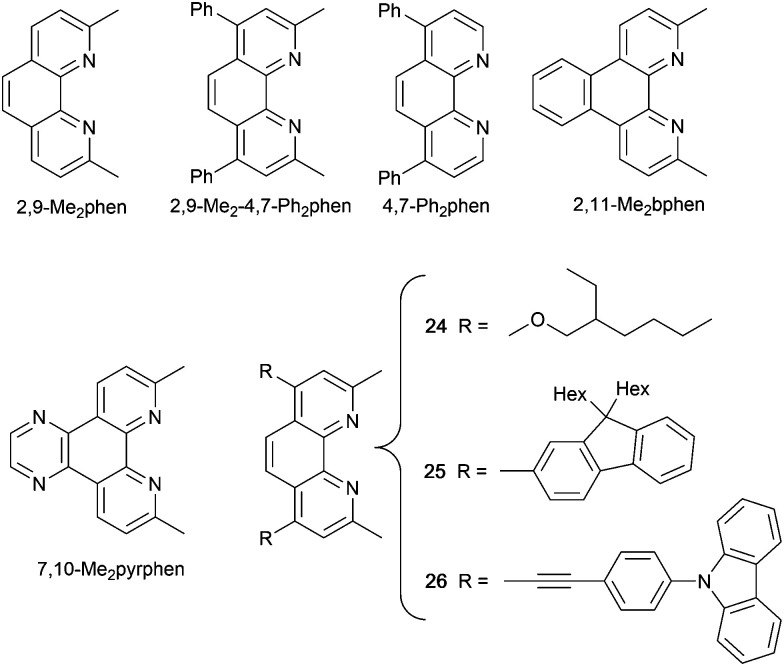
Structures of phen-based ligands combined with [dppnc]^−^ (see Scheme 10) in heteroleptic copper(i) complexes.

Several [Cu(dppnc)(N^N)] compounds have been reported with the 7,8-dicarba-*nido*-undecaborate [dppnc]^−^ (Scheme 10). Although these are neutral complexes, we include them because of the promising PL and EL characteristics of complexes which are stucturally related to [Cu(POP/xantphos/HN-xantphos)(N^N)]^+^ cations. The PL behaviours in solution, thin-film and solid and at 298 and 77 K of [Cu(dppnc)(6,6′-Me_2_bpy)], [Cu(dppnc)(phen)], [Cu(dppnc)(2,9-Me_2_phen)], [Cu(dppnc)(2,9-Me_2_-4,7-Ph_2_phen)] (Fig. 20b) and [Cu(dppnc)(4,7-Ph_2_phen)] (see Scheme 11 for phen ligands) were studied in detail, including by time-resolved emission spectroscopy. As previously discussed, the presence of the methyl substituents in the 6,6′-positions in bpy or 2,9-positions in phen are essential for preventing large structural distortions in the excited state and consequential non-radiative decay. [Cu(dppnc)(6,6′-Me_2_bpy)], [Cu(dppnc)(2,9-Me_2_phen)] and [Cu(dppnc)(2,9-Me_2_-4,7-Ph_2_phen)] are TADF emitters, and solution-processed OLEDs using [Cu(dppnc)(2,9-Me_2_phen)] and [Cu(dppnc)(2,9-Me_2_-4,7-Ph_2_phen)] as emitting dopants gave maximum EQEs of 16.57 and 15.64%, respectively. A combination of the orange emitting [Cu(dppnc)(2,9-Me_2_-4,7-Ph_2_phen)] and a Zn(ii) blue-emitter was used to achieve a white OLED with an EQE_max_ of 6.88%.^131^ The enhanced PL on going from [Cu(dppnc)(6,6′-Me_2_bpy)] to [Cu(dppnc)(2,9-Me_2_phen)] inspired further extension of the π-conjugation in the N^N domain as well as a theoretical investigation and comparison of [Cu(dppnc)(6,6′-Me_2_bpy)], [Cu(dppnc)(2,9-Me_2_phen)], [Cu(dppnc)(2,11-Me_2_bphen)] and [Cu(dppnc)(7,10-Me_2_pyrphen)] (see Scheme 11 for N^N ligands). The additional rigidity of the phen unit compared to bpy leads to a higher allowedness of the S_1_ → S_0_ transition, greater efficiency of RISC (Fig. 2) and a higher stability of the T_1_ state; all factors lead to favourable TADF. Of the compounds studied, [Cu(dppnc)(7,10-Me_2_pyrphen)] proved to exhibit the best combination of small Δ*E*_ST_ (1380 cm^−1^, 0.17 eV), high and low rates, respectively, of fluorescence and phosphorescence decays, fast RISC, and a short lifetime of the delayed fluorescence. Zou and coworkers recommend [Cu(dppnc)(7,10-Me_2_pyrphen)] as a candidate for synthesis.^132^

Judicious functionalization of the phen-based N^N ligands 24–26 (Scheme 11) resulted in [Cu(dppnc)(N^N)] complexes with EL in solution-processed OLEDs spanning from green for [Cu(dppnc)(24)] to red for [Cu(dppnc)(26)]. Although the differences in [Cu(dppnc)(24)], [Cu(dppnc)(25)] and [Cu(dppnc)(26)] lie in peripheral functionalities and, therefore, the Cu(i) coordination environment is essentially the same in each compound, the solution emission lifetimes vary from 1.0 μs for [Cu(dppnc)(26)] to 5.5 μs for [Cu(dppnc)(24)]. Che and coworkers relate this difference to the values of Δ*E*_ST_ which are calculated to be 1121 cm^−1^ (0.14 eV) for [Cu(dppnc)(24)], 1629 cm^−1^ (0.20 eV) for [Cu(dppnc)(25)] and 2073 cm^−1^ (0.26 eV) for [Cu(dppnc)(26)]. The Δ*E*_ST_ separations are therefore in line with TADF, which is further supported by extended solid-state decay lifetimes on going from 298 to 77 K, *e.g.* 15.3 to 1145.7 μs for [Cu(dppnc)(24)]. The compounds were incorporated as dopants in solution-processed OLEDs, and the green-emitting device with [Cu(dppnc)(24)] achieved an EQE of 15.20%.^133^

The aminophosphane derivatives P_2_pip and P_2_Me_2_en (Scheme 10) have been incorporated into the emissive compounds [Cu(P_2_pip)(phen)][BF_4_] and [Cu(P_2_Me_2_en)(phen)][BF_4_]. At 298 K, values of *λ*^em^_max_(PL) are 573 and 617 nm, respectively, and a red-shift is observed on going to 77 K (to 587 and 647 nm, respectively), consistent with TADF. This is also supported by the temperature dependence of the decay lifetimes. No device data were reported.^134^

## Mononuclear [Cu(P)(tripodal-N_3_)]^+^ and [Cu(P)(N^N)(N)]^+^

While [Cu(P^P)(N^N)]^+^ compounds dominate the families of Cu-iTMC TADF emitters investigated to date, variations on this coordination pattern also lead to some promising emissive materials, with TADF being established in a number of cases. In this section, we summarize progress made with [Cu(P)(tripodal-N_3_)]^+^ and [Cu(P)(N^N)(N)]^+^ coordination motifs, and in the next section, we look at softer donor sets involving sulfur.

**Scheme 12:**
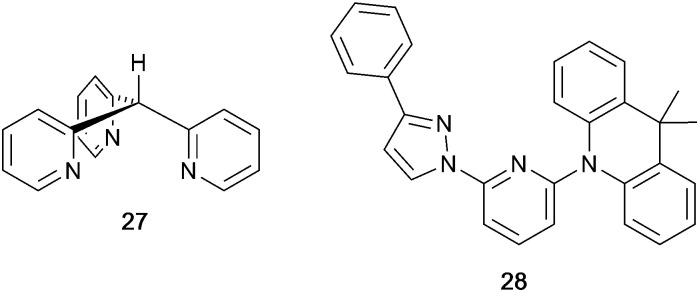
Structures of the tripodal ligand 27 and bidentate ligand 28.

**Fig. 21:**
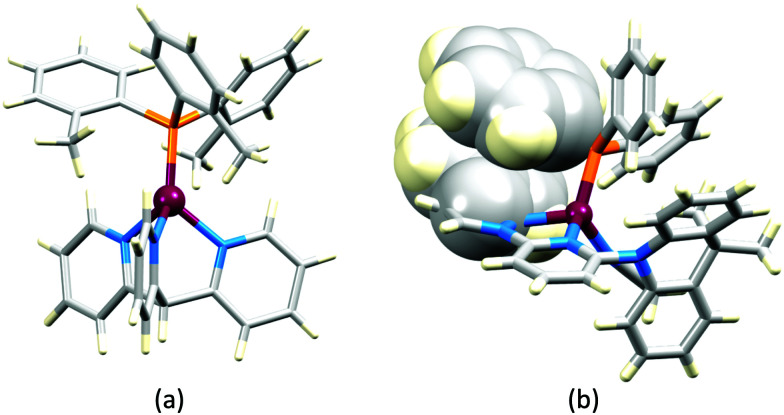
(a) Structure of the cation in [Cu{P(2-MeC_6_H_4_)_3_}(27)][PF_6_] (CSD refcode HIRJEA). (b) The structure of [Cu(PPh_3_)(28)(NCMe)]^+^ in the [BF_4_]^−^ salt (refcode NAVFUO) showing π-stacking between one phenyl ring of PPh_3_ and the phenyl ring of 28.

Earlier, we described the PL and EL behaviours of [Cu_2_(triphos)_2_(μ-4,4′-bpy)][BF_4_]_2_ (Fig. 3).^49^ This complex contained {Cu^I^(N)(tripodal-P_3_)} coordination motifs. In contrast, {Cu^I^(P)(tripodal-N_3_)} motifs are represented in [Cu(PPh_3_)(27)][X] (X^−^ = PF_6_^−^, BF_4_^−^ and BPh_4_^−^)^135^ and [Cu(PAr_3_)(27)][PF_6_] (Ar = Ph, 2-MeC_6_H_4_, 2-^*n*^BuC_6_H_4_) (see Scheme 12 for 27).^136^ These are deep-blue emitters, and in the first series, the counter-ion has a significant impact on the solid-state emission properties of [Cu(PPh_3_)(27)][X]. TD-DFT calculations show that Δ*E*_ST_ is 810 cm^−1^ (0.10 eV) for [Cu(PPh_3_)(27)]^+^, consistent with TADF behaviour. Indeed, red-shifted emission maxima are observed for powder samples on going from 300 to 77 K (466 to 478 nm for X^−^ = PF_6_^−^, 449 to 462 nm for X^−^ = BF_4_^−^, 452 to 462 nm for X^−^ = BPh_4_^−^) and decay lifetimes increase (14 to 26 μs, 7.5 to 19 μs, and 5.4 to 25 μs for PF_6_^−^, BF_4_^−^ and BPh_4_^−^ salts, respectively). The counter-ion also affects the solid-state PLQYs with 43% for [Cu(PPh_3_)(27)][PF_6_] and 7% for [Cu(PPh_3_)(27)][BPh_4_].^135^ Changing the aryl groups in [Cu(PAr_3_)(27)][PF_6_] from Ph to 2-MeC_6_H_4_ or 2-^*n*^BuC_6_H_4_ has a dramatic effect on the PLQY, even in solution. Upon excitation, [Cu(PPh_3_)(27)]^+^ undergoes significant distortion leading to non-radiative deactivation and emission quenching; the solution PLQY for [Cu(PPh_3_)(27)][PF_6_] is <1%. When the steric demands of PAr_3_ increase, the non-radiative pathways decrease, and the solution PLQY values increase dramatically to 58% for [Cu{P(2-MeC_6_H_4_)_3_}(27)][PF_6_], and 76% for [Cu{P(2-^*n*^BuC_6_H_4_)_3_}(27)][PF_6_]. Fig. 21a shows the structure of the [Cu{P(2-MeC_6_H_4_)_3_}(27)]^+^ cation, and illustrates that the Me substituents of the tolyl groups provide additional steric protection for the Cu(i) centre. On going from CH_2_Cl_2_ solutions of [Cu(PAr_3_)(27)][PF_6_] to powders, *λ*^em^_max_(PL) blue-shifts, and a blue-shift is also seen along the series PPh_3_ to P(2-MeC_6_H_4_)_3_ or P(2-^*n*^BuC_6_H_4_)_3_. Both trends are consistent with greater rigidity of the system. The solid-state PLQY is highest for [Cu{P(2-MeC_6_H_4_)_3_}(27)][PF_6_] (86%). A thorough investigation of this complex reveals a notably fast phosphorescence decay rate (5 × 10^4^ s^−1^) and although TADF takes effect above 160 K, phosphorescence of [Cu{P(2-MeC_6_H_4_)_3_}(27)][PF_6_] prevails over TADF (60% *vs.* 40%) at 298 K.^136^ This is a very promising family of Cu-iTMCs and one that deserves further investigations in emitting devices.

In [Cu(PPh_3_)(28)(NCMe)][BF_4_], the crystal structure reveals inter-ligand π-stacking (Fig. 21b) and significant steric protection of the Cu(i) centre. The powdered compound exhibits a blue-green emission (*λ*^em^_max_(PL) = 492 nm) with PLQY = 27.9% and *τ* = 235 μs, and it is proposed that this originates from ILCT excited states. The variable temperature emission behaviour supports a TADF mechanism at ambient temperatures.^137^

## Mononuclear [Cu(P^P)(N^S)]^+^ and [Cu(P^P)(P^S)]^+^

**Scheme 13:**
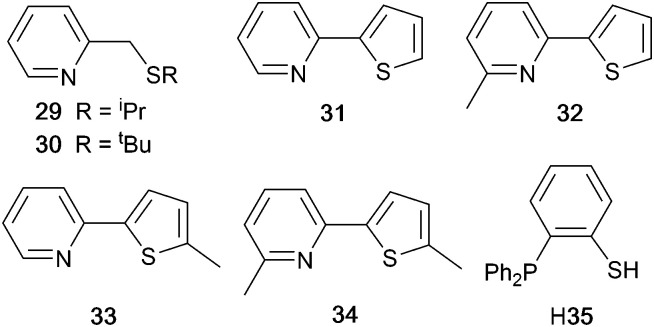
Structures of chelating N^S and P^S ligands.

We recently investigated the effects of replacing the N^N donor set by the N^S ligands 29–34 (Scheme 13). [Cu(P^P)(29)][PF_6_] and [Cu(P^P)(30)][PF_6_] with P^P = POP and xantphos^138^ and [Cu(POP)(N^S)][PF_6_] with N^S = 31, 32, 33 and 34^139^ are very weakly emissive in the solid state. Powdered samples of [Cu(xantphos)(N^S)][PF_6_] with N^S = 31, 32, 33 and 34 are yellow emitters with PLQYs in the range 4.7–10.8%, the highest value being for [Cu(xantphos)(31)][PF_6_]. This compound was incorporated as the electroluminophore in a LEC, but exhibited poor EL and poor charge transporting properties.^139^

The neutral complex [Cu(dppb)(35)] (dppb = 1,2-bis(diphenylphosphano)benzene, H35 is shown in Scheme 13) is a green emitter. In the solid state, *λ*^em^_max_(PL) = 521 nm and PLQY = 52% at 293 K, and the corresponding values at 77 K are 534 nm and 73%, with an increased decay lifetime on cooling. With a value of Δ*E*_ST_ of 309 cm^−1^ (0.038 eV), [Cu(dppb)(35)] shows efficient TADF behaviour at room temperature. The strong electron-donating character of the [35]^−^ ligand reduces the Cu contribution to the highest occupied MOs of [Cu(dppb)(35)], and as a result, LLCT rather than MLCT character in the excited states becomes important. Solution-processed OLEDs with an active layer comprising 4,4′-bis(9-carbazolyl)-2,2′-dimethylbiphenyl doped with 10% [Cu(dppb)(35)] were fabricated. Additional doping with di-[4-(*N*,*N*-ditolylamino)phenyl]cyclohexane contributed to the best OLED current efficiency of 21.3 cd A^−1^ and EQE_max_ of 7.8%.^140^

## Di- and polynuclear complexes with P^P, N^N and P^S metal-binding domains

**Scheme 14:**
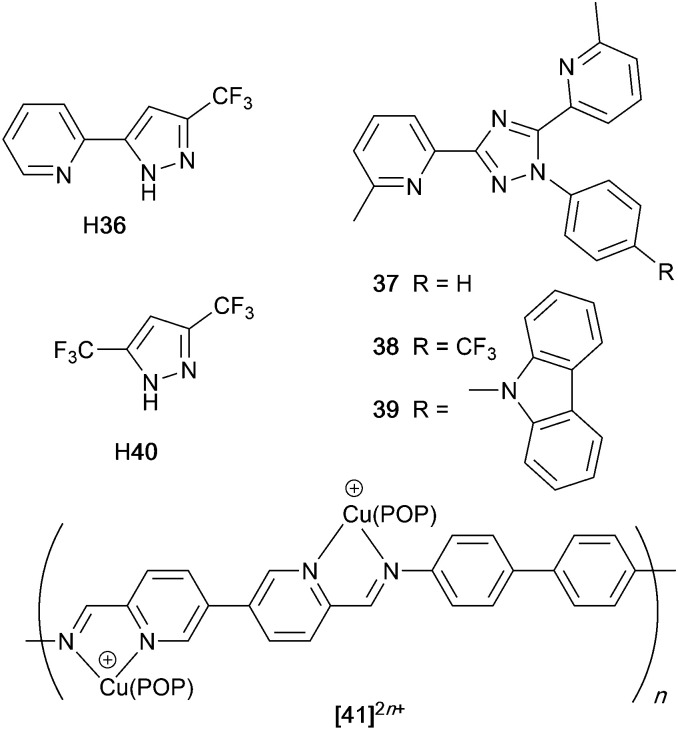
Structures of some N^N or bis(N^N) ligands in dinuclear Cu(i) complexes, and the structure of metallopolymer [41]^2*n*+^.

We first consider species in which the Cu(i) centres are bridged by bis- or tetrakisphosphano ligands. We have already discussed the dinuclear complexes [Cu_2_(20)_2_(μ-POP)]^2+^ (a rare example in which POP is in a bridging mode) and [Cu_2_(POP)_2_(μ-22)]^2+^ (in which the sulfone 22 coordinates to two Cu(i) centres through two N,O-donor sets).^121^ A number of dinuclear complexes feature N^N ligands of the types previously discussed and bridging bisphosphanes. [Cu_2_(2,9-Me_2_phen)_2_(μ-dppa)_2_][BF_4_]_2_ (dppa = bis(diphenylphosphano)ethyne) is poorly emissive in solution (PLQY = 1%); in a PMMA film at 298 K, the PLQY is 6% and this increases to 56% at 77 K. However, the emission maximum at 77 K has vibrational structure, indicative of ^3^ππ* character of the emission, and this behaviour for the dinuclear species contrasts with that of the related mononuclear [Cu(xantphos)(2,9-Me_2_phen)]^+^ which is a TADF emitter (see earlier).^105^ [Cu_2_(36)_2_(μ-dppe)_2_] (H36 is shown in Scheme 14, dppe = bis(diphenylphosphano)ethane) is unusual in that the CH_2_Cl_2_ solvate exhibits luminescent vapochromism with a yellow to cyan PL colour change; when the desolvated crystalline material is exposed to CH_2_Cl_2_ vapour, PL cyan emission returns to a yellow emission. The effect is selective to CH_2_Cl_2_ and is attributed to the accommodation of each CH_2_Cl_2_ molecule within a pair of pyridine rings of adjacent [Cu_2_(36)_2_(μ-dppe)_2_] complexes in the lattice. The desolvated solid [Cu_2_(36)_2_(μ-dppe)_2_] has *λ*^em^_max_(PL) = 493 nm at room temperature and the emission shifts to 509 nm at 77 K, with the value of *τ* increasing from 24 μs at 350 K to 164 μs at 50 K. These data and the value of Δ*E*_ST_ = 0.097 eV (780 cm^−1^) are consistent with TADF behaviour. [Cu_2_(36)_2_(μ-dppe)_2_] is readily sublimable and was incorporated into vapour-deposited multilayer OLEDs with 4,4′-bis(9-carbazolyl)-2,2′-dimethylbiphenyl as the host material in the active layer. Of the doping concentrations used, the best OLED performance was found for 6 wt% of [Cu_2_(36)_2_(μ-dppe)_2_] with values of Lum_max_ = 7217 cd m^−2^ and EQE_max_ = 7.5%.^141^

**Fig. 22:**
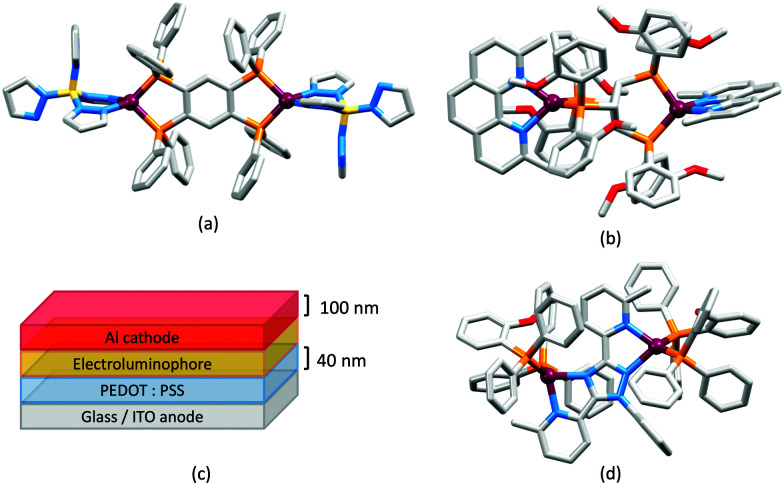
Structures of (a) [Cu_2_(pz_4_B)_2_(μ-tpbz)] (CSD refcode KURCAD), and (b) the cationic complex in [Cu_2_(2,9-Me_2_phen)_2_(μ-MeOdppcb)][PF_6_]_2_ (refcode VOZQAE). (c) Architecture of a LEC containing [Cu_2_(2,9-Me_2_phen)_2_(μ-MeOdppcb)][PF_6_]_2_ in the active layer; the LEC was driven under a bias of 4 or 5 V. (d) Structure of the dinuclear cation in [Cu_2_(POP)_2_(μ-37)][BF_4_]_2_ (refcode MAMXUW). H atoms are omitted from the figures for clarity.

Earlier, we described the seminal work of Yersin and coworkers in which the TADF behaviour of [Cu(POP)(pz_2_BH_2_)], [Cu(POP)(pz_2_BPh_2_)] and [Cu(POP)(pz_4_B)] (Fig. 6 and associated discussion) was revealed.^62^ Related dinuclear compounds are [Cu_2_(pz_4_B)_2_(μ-tpbz)] (Fig. 22a) (tpbz = 1,2,4,5-tetrakis(diphenylphosphano)benzene), [Cu_2_(pz_2_BH_2_)_2_(μ-tpbz)] and [Cu_2_(tz_2_BH_2_)_2_(μ-tpbz)] ([tz_2_BH_2_]^−^ = bis(1,2,4-triazol-1-yl)borohydrate). Solids samples exhibit yellow-orange PL with *λ*^em^_max_(PL) = 580, 569 and 540 nm, respectively, with PLQYs of 7, 28 and 45%, respectively, at 298 K. Extended decay lifetimes upon going from 298 to 77 K are observed (*e.g.* 11.9 to 29.1 μs for [Cu_2_(pz_2_BH_2_)_2_(μ-tpbz)]). With estimated values of Δ*E*_ST_ in the range 0.06 and 0.09 eV (*ca.* 500–700 cm^−1^, corrobated by TD-DFT calculations), the compounds are likely to be TADF emitters at ambient temperatures. However, atypically for TADF, the emission maxima undergo small blue-shifts of between 6 and 10 nm on going from 298 to 77 K.^142^ 1,2,3,4-Tetrakis(diphenylphosphano)cyclobutane (dppcb) and 1,2,3,4-tetrakis{di(2-methoxyphenyl)phosphano}cyclobutane (MeOdppcb) act as bis(chelating) ligands in [Cu_2_(5,5′-Me_2_bpy)_2_(μ-dppcb)][PF_6_]_2_, [Cu_2_(2,9-Me_2_phen)_2_(μ-MeOdppcb)][PF_6_]_2_ and [Cu_2_(15)_2_(μ-MeOdppcb)] (see Scheme 8 for H15). In terms of emission behaviour, [Cu_2_(2,9-Me_2_phen)_2_(μ-MeOdppcb)][PF_6_]_2_ is the most interesting of this series, exhibiting TADF behaviour. The combined steric effects of 2,9-Me_2_phen and the P^P metal-binding domain (Fig. 22b) contribute to a PLQY of 49% for a deaerated MeCN solution (*λ*^em^_max_ = 554 nm, *τ* = 13.8 μs at 298 K). The emission is red-shifted on going to 77 K, and *τ* increases to 634 μs. [Cu_2_(2,9-Me_2_phen)_2_(μ-MeOdppcb)][PF_6_]_2_ was used in the active layer of a simple LEC (Fig. 22c) and the EL maximum corresponds to the *λ*^em^_max_(PL) of a pristine thin-film (*ca.* 554 nm). Under a driving voltage of 5 V, the LEC had a fast a turn-on to reach a Lum_max_ of 108 cd m^−2^. However, the decay of the EL was rapid. A longer EL lifetime was achieved at the expense of brightness with a 4 V driving voltage. This contribution from Brüggeler and De Cola in 2014 is noteworthy for being a relatively early report of TADF heteroleptic copper(i) emitters proven to function in LECs.^143^

**Fig. 23:**
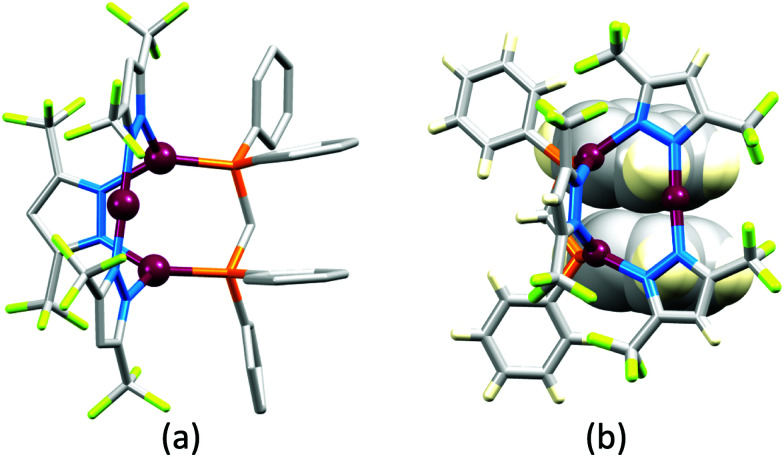
(a) Structure of [Cu_3_(dppm)(40)_3_] (CSD refcode NOGZOB) with H atoms omitted, and (b) view of the same molecule showing the π-stacking within the dppm ligand.

We now move to polynuclear copper(i) complexes featuring bridging N-donor ligands. The majority are neutral compounds and are included because of some notable PL and device performances. [Cu_2_(POP)_2_(μ-37)_2_] (Fig. 22d), [Cu_2_(POP)_2_(μ-38)_2_] and [Cu_2_(POP)_2_(μ-39)_2_] differ in the functionalization of a phenyl ring in the bis(N^N) ligand (Scheme 14). As is typical, relatively low solution PLQYs are enhanced on going to a more rigid matrix. In PMMA films (20% weight concentration), PLQYs of 20, 16 and 26% (*λ*^em^_max_(PL) = 514, 506 and 512 nm) for [Cu_2_(POP)_2_(μ-37)_2_], [Cu_2_(POP)_2_(μ-38)_2_] and [Cu_2_(POP)_2_(μ-39)_2_], respectively, are observed, and further improvement in PLQY is seen when moving from PMMA to PYD2 (see Fig. 15 for PYD2). The compounds are TADF emitters at ambient temperatures. On going from 298 to 77 K, values of *λ*^em^_max_(PL) for solid [Cu_2_(POP)_2_(μ-37)_2_], [Cu_2_(POP)_2_(μ-38)_2_] and [Cu_2_(POP)_2_(μ-39)_2_] red-shift from 509, 519 and 503 nm, respectively, to 523, 546 and 516 nm, and decay lifetimes increase (5.5, 16 and 5.5 μs to 158, 356 and 209 μs, respectively). The experimentally determined S_1_–T_1_ separations, Δ*E*_ST_, are 0.089, 0.132 and 0.094 eV (*ca.* 700, 1060, 800 cm^−1^) for [Cu_2_(POP)_2_(μ-37)_2_], [Cu_2_(POP)_2_(μ-38)_2_] and [Cu_2_(POP)_2_(μ-39)_2_], respectively. The promising PL properties of these compounds led to them being used as electroluminophores in solution-processed, multilayer OLEDs with PYD2 (see Fig. 15) as the host material. The OLED with [Cu_2_(POP)_2_(μ-39)_2_] exhibited the highest values of EQE (8.3%) and Lum_max_ (2525 cd m^−2^), with the hole-transporting properties of the carbazole group in 39 contributing to the performance.^144^ In 2019, Titov *et al.* reported the first example of a cyclic tricopper(i) pyrazolate displaying TADF behaviour.^145^ [Cu_3_(dppm)(40)_3_] (Scheme 14 shows H40, and dppm = bis(diphenylphosphano)methane) contains two Cu(i) centres bridged by both N^N and P^P domains and one two-coordinate Cu(i) bound only by N-donors (Fig. 23a). The authors comment that the photophysical properies are influenced by intramolecular structural features rather than by intermolecular interactions. It is worth noting, therefore, that the crystal structure of [Cu_3_(dppm)(40)_3_] exhibits π-stacking between two Ph rings of dppm (Fig. 23b), reminiscent of the intra-POP interactions described earlier. On going from 298 to 77 K, the solid-state emission of [Cu_3_(dppm)(40)_3_] shifts from 514 to 554 nm, and *τ* increases from 32.7 to 148.6 μs, consistent with TADF behaviour. The value of Δ*E*_ST_ derived from experimental data is estimated to be 1080 ± 60 cm^−1^ (0.13 ± 0.01 eV). The PL properties suggest that this and related trinuclear species may find applications in lighting devices, but no relevant OLED data are yet available.

The metallopolymer [41][BF_4_]_2*n*_ was prepared by condensation of [3,3′-bipyridine]-6,6′-dicarbaldehyde and 1,1′-biphenyl-4,4′-diamine in the presence of Cu(BF_4_)_2_ and POP. A DMF solution of the polymer gave an emission with *λ*^em^_max_(PL) = 780 nm, but after heating at 160 °C, a yellow gel formed for which *λ*^em^_max_(PL) = 580 nm; cooling reversed the sol–gel transition. The changes were attributed to reversibie dissociation of the complex. LECs were fabricated using an architecture similar to that shown in Fig. 22c, with a 100 ± 20 nm active layer and 100 nm Al cathode layer. With an onset voltage of *ca.* 4 V, a value of Lum_max_ = 3 cd m^−2^ was reached. At low bias, the EL was in the IR region, but an increase in voltage produced a blue-shift eventually giving yellow EL. The process was reversible and, by analogy with the solgel transition, was explained in terms of reversible dissociation of [Cu(POP)]^+^ domains from the organic polymer backbone. Although TADF was not demonstrated in this system, the investigation is of relevance for this review in terms of establishing the effects of heat on PL and of higher bias in LECs containing heteroleptic copper(i) emitters.^146^

## Copper(i) complexes with N-heterocyclic carbenes

N-Heterocyclic carbenes (NHCs) are characterized by exhibiting strong σ-donating and weak π-accepting properties. The potential for employing NHC ligands in emissive three-coordinate copper(i) compounds was described by Thompson and coworkers between 2010 and 2014.^147–149^ In 2019, Danopoulos *et al.* provided a thorough overview of the field of NHC copper complexes including cyclic alkyl-amino carbenes (cAACs),^150^ and we have therefore chosen to focus on early pivotal investigations, and then on Cu-iTMCs (*i.e.* ionic complexes) which incorporate NHC ligands.

### Three-coordinate copper

**Scheme 15:**
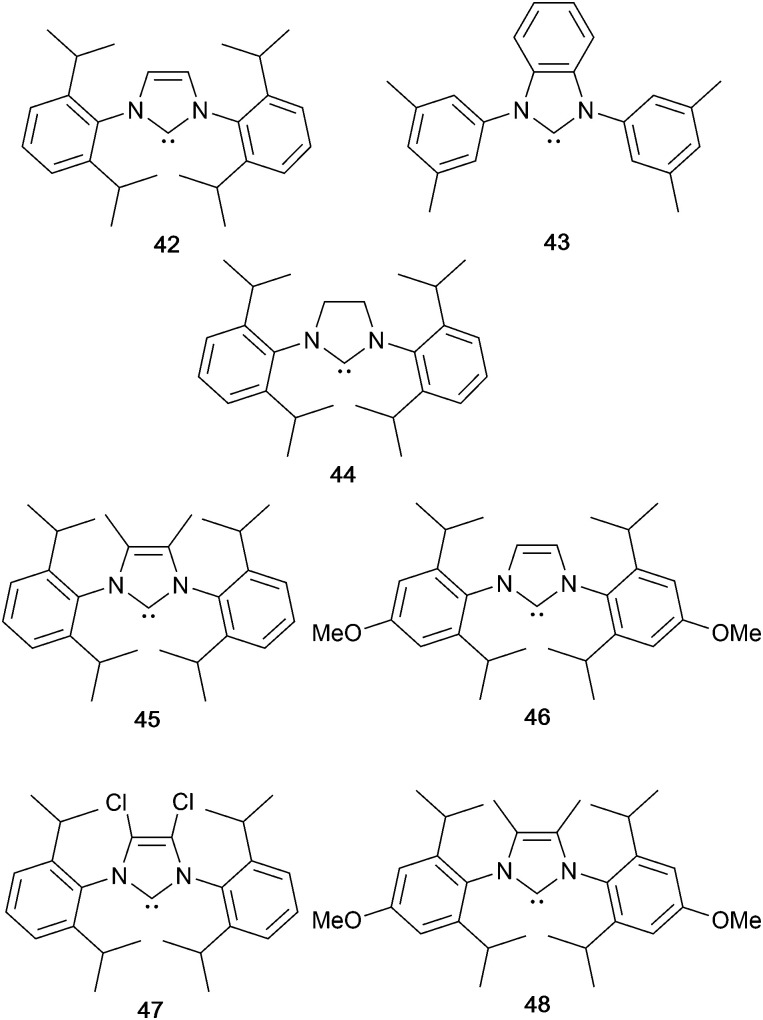
Structures of NHC ligands 42–48.

With respect to TADF, a comparison of the properties of [Cu(42)(py_2_BMe_2_)] and [Cu(43)(py_2_BMe_2_)] is highly instructive; NHCs 42 and 43 are shown in Scheme 15, and [py_2_BMe_2_]^−^ (dimethyldi(pyridin-2-yl)borate) acts as an N^N ligand in an analogous fashion to [pz_2_BH_2_]^−^ (see Fig. 6). At 300 K, solid [Cu(42)(py_2_BMe_2_)] and [Cu(43)(py_2_BMe_2_)] are blue and yellow emitters, respectively, with *λ*^em^_max_(PL) = 475 and 575 nm and PLQYs of 76 and 73%. At 77 K, *λ*^em^_max_(PL) = 490 and 585 nm, respectively, and values of *τ* are 34 and 21 μs, compared to 11 and 18 μs at 300 K. For [Cu(42)(py_2_BMe_2_)], Δ*E*_ST_ is 740 cm^−1^ (0.092 eV) allowing this complex to exhibit TADF behaviour at ambient temperatures. [Cu(42)(py_2_BMe_2_)] shows two radiative decay paths: 62% TADF *vs.* 38% phosphorescence. In contrast, on going from 42 to 43, expanding the conjugation and replacing the 2,6-isopropyl by 3,5-dimethyl groups lead to a significantly increased value of Δ*E*_ST_ in [Cu(43)(py_2_BMe_2_)] (3000 cm^−1^, 0.37 eV) which militates against RISC and, therefore, TADF. The different N_NHC_–C_NHC_–Cu–N_py_ torsion angles of 5° in [Cu(42)(py_2_BMe_2_)] *vs.* 70° in [Cu(43)(py_2_BMe_2_)] are key to the different photophysical behaviours, and lay the foundations for rational structural design within this family of copper(i) emitters.^39^ Thus, NHCs containing 2,6-isopropylphenyl substituents (*e.g.*42, 44–48 in Scheme 15) are popular choices for NHC copper(i) complexes.

**Scheme 16:**
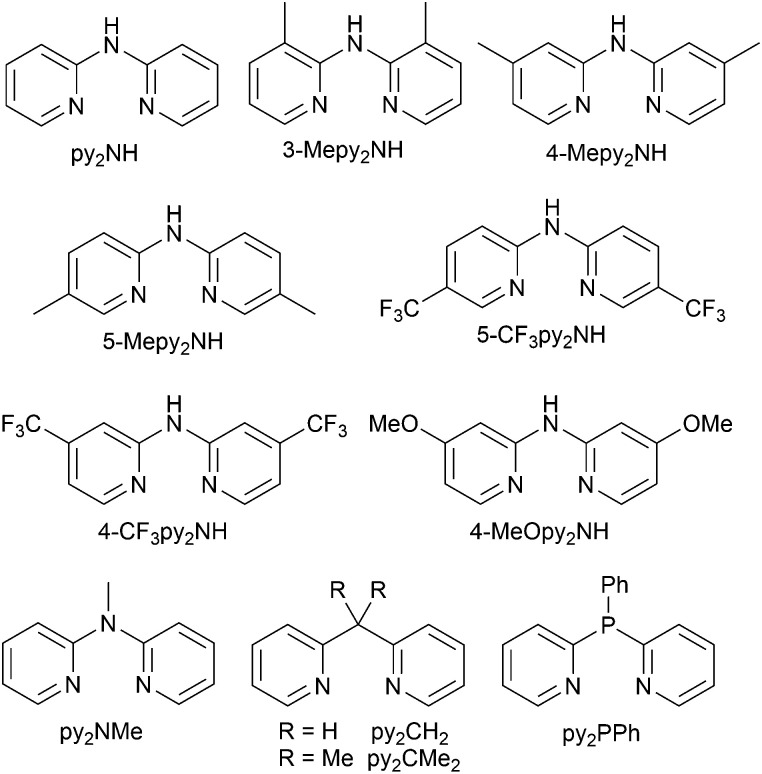
Structures of di(pyridin-2-yl)amine N^N ligands.

**Fig. 24:**
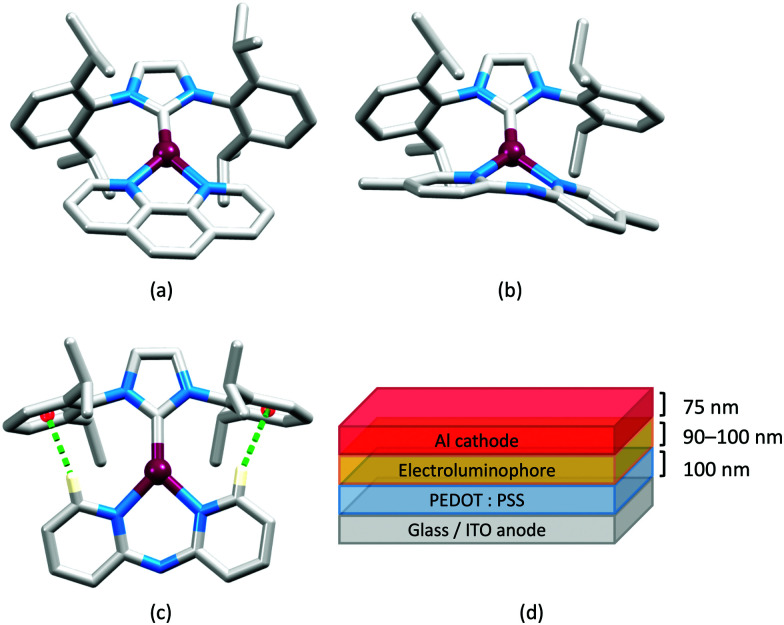
Comparison of the structures of the [Cu(N^N)(NHC)]^+^ cations in (a) [Cu(phen)(42)][BF_4_] (CSD refcode SORNOE) and (b) [Cu(5-Mepy_2_NH)(42)][PF_6_] (refcode SORPIA). (c) Intramolecular CH⋯π interactions in [Cu(py_2_NH)(42)][PF_6_] (refcode SORPOG). (d) Architecture of the LECs containing Cu-iTMCs with NHC and py_2_NH-type N^N ligands; LECs were driven using a pulsed current.

Replacement of the [BMe_2_]^−^ unit in [py_2_BMe_2_]^−^ by CR_2_, NR or PR leads to neutral N^N ligands and an entry into NHC-containing Cu-iTMCs for LECs. This strategy complements the investigations of the luminescent three-coordinate complexes [Cu(phen)(42)]^+^,^147,151,152^ [Cu(bpy)(42)]^+^ and [Cu(4,4′-Me_2_bpy)(42)]^+^.^151,152^ Attempts to isolate [Cu(2,9-Me_2_phen)(42)][PF_6_] led only to the homoleptic complexes [Cu(2,9-Me_2_phen)_2_][PF_6_] (four-coordinate Cu) and [Cu(42)_2_][PF_6_] (two-coordinate Cu), indicating that the combined steric demands of the Me groups in 2,9-Me_2_phen and the ^*i*^Pr substituents in 42 are too great for a three-coordinate Cu(i) centre.^151^ [Cu(phen)(42)][OTf],^147^ [Cu(phen)(42)][PF_6_], [Cu(bpy)(42)][PF_6_] and [Cu(4,4′-Me_2_bpy)(42)][PF_6_]^151^ are weakly emissive in solution, with some enhancement seen for [Cu(phen)(42)][OTf] in frozen 2-MeTHF at 77 K.^147^ In contrast, use of di(pyridin-2-yl)amines py_2_NH, 3-Mepy_2_NH, 4-Mepy_2_NH and 5-py_2_NH (Scheme 16) lead to the respective [Cu(N^N)(42)][PF_6_] complexes which are blue-emitters in the solid state (*λ*^em^_max_(PL) = 436–488 nm) with PLQYs in the range 5–86%, the highest being for [Cu(3-Mepy_2_NH)(42)][PF_6_]. Decay lifetimes are between 17 and 44 μs.^151,152^ The emission properties of [Cu(py_2_NH)(44)][PF_6_] (*λ*^em^_max_(PL) = 484 nm, PLQY = 88%, *τ* = 51 μs) are similar to those of [Cu(3-Mepy_2_NH)(42)][PF_6_]. This series of compounds represented the first [Cu(N^N)(NHC)]^+^ species with high PLQYs and blue emissions. Critically, use of a di(pyridin-2-yl)amine rather than a planar (*e.g.* bpy or phen) N^N domain modifies the structure (Fig. 24) such that there is an increase in the HOMO–LUMO separation. An important structural feature of this series of heteroleptic complexes is intramolecular, inter-ligand CH⋯π interactions between pyridine CH and *N*-phenyl substituents (Fig. 24c).^151^ This interaction appears to be especially important in improving the air and moisture stability of the compounds in the solid state.^153^ The investigations of [Cu(N^N)(NHC)]^+^ Cu-iTMCs were extended to a wider range of NHC (Scheme 15) and py_2_NH-type N^N (Scheme 16) ligands in order to establish structure–property relationships and to demonstrate TADF in three-coordinate [Cu(N^N)(NHC)]^+^ complexes.^154^ In respect of the N^N ligands, the presence of electron-donating groups (Me, OMe) leads to a blue-shifted emission, whereas electron-withdrawing groups (CF_3_) cause a red-shift; going from py_2_NH to py_2_NMe leads to a small blue-shift in *λ*^em^_max_(PL) of [Cu(N^N)(NHC)]^+^. A correlation between the presence of H_N^N_⋯F_anion_ contacts and enhanced PLQY was also proposed. It was also noted that along the series [Cu(py_2_NH)(NHC)][PF_6_] in which NHC = 42, 45, 46, 47 and 48 (Scheme 15), an increase of the σ-donation of the NHC corresponds to an increase in the solid-state PLQY (*e.g.* 17% for [Cu(py_2_NH)(47)][PF_6_]), 22% for [Cu(py_2_NH)(42)][PF_6_] and 64% for [Cu(py_2_NH)(48)][PF_6_]. This correlates to a trend in the Cu–C_NHC_ bond length: the shorter the Cu–C_NHC_, the higher the PLQY. LECs have been fabricated with the configuration shown in Fig. 24d with [Cu(py_2_NH)(42)][PF_6_], [Cu(py_2_NH)(48)][PF_6_], [Cu(4-CF_3_py_2_NH)(42)][PF_6_] or [Cu(3-Mepy_2_NH)(42)][PF_6_] in the active layer. LECs with [Cu(py_2_NH)(42)][PF_6_] and [Cu(3-Mepy_2_NH)(42)][PF_6_] exhibited Lum_max_ = 56 and 310 cd m^−2^, respectively, and use of [Cu(3-Mepy_2_NH)(42)][PF_6_] provided the first blue-emitting copper-based LEC (*λ*^em^_max_(EL) = 490–500 nm). Gaillard, Costa and coworkers note that one difficulty is that the [Cu(py_2_NH)(NHC)][PF_6_] complexes slowly degrade in solution (see later).^154^

**Fig. 25:**
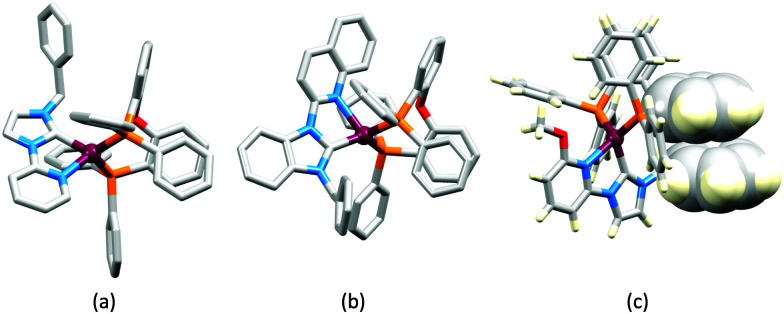
Structures of (a) the [Cu(POP)(49)]^+^ cation, and (b) the [Cu(POP)(50)]^+^ cation in their [PF_6_]^−^ salts (CSD refcodes QAKPIE and QAKPUQ). H atoms are omitted for clarity. (c) Structure of the [Cu(POP)(52)]^+^ cation in the [PF_6_]^−^ salt (refcode ZIKTEV) showing (space-filling representation) the π-stacking between the Ph ring in 52 and one arene ring of the POP backbone; there is a second π-stacking contact within the POP ligand (top of figure).

Gaillard, Costa and coworkers have extended the use of py_2_NH-type ligands to py_2_CH_2_, py_2_CMe_2_ and py_2_PPh (Scheme 16), each combined with NHC 42 in [Cu(N^N)(NHC)][PF_6_] salts. Along the series [Cu(N^N)(42)][PF_6_], the solid-state emission *λ*^em^_max_(PL) at 298 K is red-shifted for N^N = py_2_CH_2_, py_2_CMe_2_ and py_2_PPh (473, 474 and 503 nm, respectively) compared to 463 nm for py_2_NH. PLQYs are lowest for [Cu(py_2_CH_2_)(42)][PF_6_] (15%) and highest for [Cu(py_2_PPh)(42)][PF_6_] (86%). On going from 298 to 77 K, all compounds show a red-shifted emission and extended *τ* values, *e.g.* 503 to 519 nm, and 13 to 87 μs for [Cu(py_2_PPh)(42)][PF_6_], consistent with TADF. The nature of the bridging group in the py_2_X ligand does not have a significant effect on Δ*E*_ST_, values of which were determined as 0.095 eV (*ca.* 760 cm^−1^) for [Cu(py_2_NH)(42)][PF_6_], 0.12 eV (*ca.* 1000 cm^−1^) for [Cu(py_2_CH_2_)(42)][PF_6_], 0.10 eV (*ca.* 800 cm^−1^) for [Cu(py_2_CMe_2_)(42)][PF_6_], and 0.10 eV (*ca.* 800 cm^−1^) for [Cu(py_2_PPh)(42)][PF_6_]. This series of TADF-emitters was incorporated into LECs with an architecture similar to that shown in Fig. 25d, but with layer thicknesses of 70 nm PEDOT:PSS, 90 nm electroluminophore, and 90 nm Al. Compared to the complexes containing py_2_NH and py_2_CH_2_, [Cu(py_2_CMe_2_)(42)][PF_6_] and [Cu(py_2_PPh)(42)][PF_6_] showed enhanced ionic mobilities which allowed the LECs to be driven under lower pulsed currents. Fast turn-on with values of Lum_max_ = 6.2 and 13 cd m^−2^ and efficacy = 0.19 and 0.39 cd A^−1^, respectively, were achieved at the lowest applied current (0.5 mA).^155^ The development of NHC Cu-iTMCs for blue- and green-emitting LECs has gained momentum, but as previously mentioned, degradation of [Cu(N^N)(NHC)]^+^ species during solution processing is problematical. An important contribution which addesses device optimization demonstrates the use of ionic additives, as well as a hole transporter. With such modifications, the Lum_max_ of LECs containing [Cu(py_2_NH)(42)][PF_6_] could be boosted from 20 to 160 cd m^−2^, and the efficiency from 0.17 to 1.2 cd A^−1^.^153^

### Two-coordinate copper

In 2019, Marian and coworkers reported the TADF behaviour of a series of two-coordinate Cu-iTMCs. Initial findings were that [Cu(py)(42)][BF_4_], [Cu(2-Mepy)(42)][BF_4_] and [Cu(2-Phpy)(42)][BF_4_] (py = pyridine, 2-Mepy = 2-methylpyridine, 2-Phpy = 2-phenylpyridine) were poorly or non-emissive in single crystal form or in solution, although in powder form or thin films, the cation–anion pairing through the formation of Cu–F–B interactions turned on a blue or blue-green PL.^156^ This was followed up with a theoretical investigation of [Cu(py)(42)][BF_4_], [Cu(2-Mepy)(42)][BF_4_] and [Cu(2-Phpy)(42)][BF_4_] which showed that the PL is quenched by locally excited triplet states if, as in 42, the NHC ligand carries two diisopropylphenyl substituents. Replacement of 2,6-^*i*^Pr_2_C_6_H_3_ in the NHC ligand 42 in [Cu(py)(42)]^+^ by adamantyl groups suppresses emission quenching and the theoretical study predicts that TADF will then be efficient. Further, introducing electron-withdrawing or -donating substituents into the 4-position in the py ligand should lead to emission tuning over the UV, blue and green regions.^157^ Although these results should motivate synthetic investigations, the predictions do not yet appear to have been experimentally established. Extensive calculational studies from Marian and coworkers in 2020, should provide ground rules for structure design in NHC copper(i) coordination compounds. One pertinent conclusion relevant to two-coordinate (linear) complexes is that only species with S_1_ and T_1_ states with LLCT character have sufficiently small values of Δ*E*_ST_ to facilitate TADF. Complexes in which MLCT character predominates in the S_1_ and T_1_ excited states tend to decay by phosphorescence.^41^

### Four-coordinate copper

**Scheme 17:**
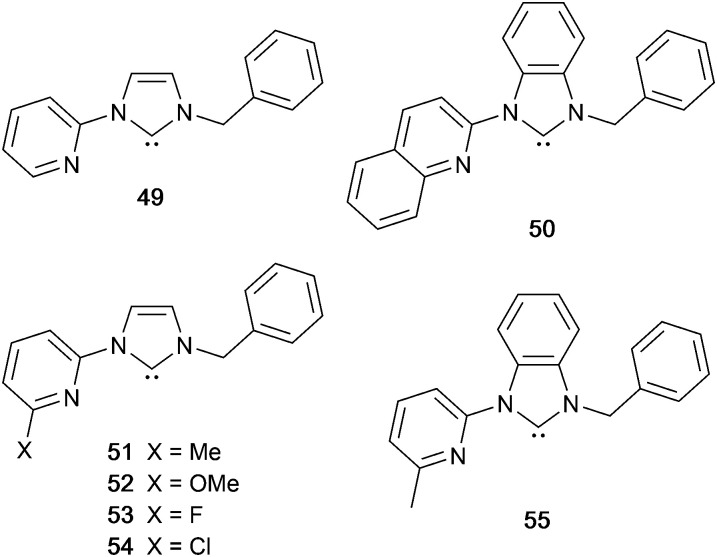
NHC ligands used in [Cu(POP)(NHC)][PF_6_] emitters.

In this final section dealing with NHC-containing Cu-iTMCs, we focus on four-coordinate [Cu(POP)(NHC)]^+^ complexes, in which the NHC ligand carries a substituent which acts as an N-donor (Scheme 17). [Cu(POP)(49)][PF_6_] and [Cu(POP)(50)][PF_6_] both contain distorted tetrahedral Cu(i) centres. Both complex cations (Fig. 25) exhibit the characteristic π-stacking interaction within the POP ligand (see earlier discussion), but there are no efficient intramolecular π-stacking contacts between the NHC and POP ligands. The Cu–C_carbene_ bonds are longer (1.966(6) and 1.957(4) Å) in [Cu(POP)(49)][PF_6_] and [Cu(POP)(50)][PF_6_] than in the typical three-coordinate [Cu(N^N)(NHC)]^+^ complexes described above, consistent with greater steric demands within the copper(i) coordination spheres in the four-coordinate species. In solution, [Cu(POP)(49)][PF_6_] and [Cu(POP)(50)][PF_6_] are non-emissive, but in the solid state they are green and yellow emitters, respectively, with PLQY values of 56 and 35%. The values of *λ*^em^_max_(PL) of 520 nm for [Cu(POP)(49)][PF_6_] and 570 nm for [Cu(POP)(50)][PF_6_] at 298 K red-shift to 553 and 612 nm, respectively, on cooling to 77 K, and values of *τ* increase. For [Cu(POP)(49)][PF_6_], the emission decay was monoexponential at 298 K, and a biexponential fit was used at 77 K. In contrast, bi- and triexponential fits were applied for the emission decays of [Cu(POP)(50)][PF_6_] at 298 and 77 K, respectively. Analysis of the solid-state emission behaviour leads to the conclusion that both compounds are predominantly TADF emitters at 298 K, with a phosphorescence component.^158^ This first contribution from Wang *et al.* focused on the effects of extending the π-conjugation in the NHC ligand (49*vs.*50).^158^ In a later investigation from the same group, the effects of introducing electron-donating Me and OMe or electron-withdrawing F or Cl substituents (NHCs 51–54) were explored.^159^ Structurally, [Cu(POP)(51)]^+^, [Cu(POP)(52)]^+^, [Cu(POP)(53)]^+^ and [Cu(POP)(54)]^+^ are similar to [Cu(POP)(49)]^+^ (all characterized as [PF_6_]^−^ salts), and exhibit the typical π-stacking contact within the POP ligand. Additionally, in the solid-state structures of the methoxy and fluoro derivatives [Cu(POP)(52)][PF_6_] and [Cu(POP)(53)][PF_6_], there is a π-stacking between the Ph ring in 52 or 53 and one arene ring of POP (Fig. 25c). These interactions are not discussed in detail by Wang *et al.*, but we highlight them here in view of the known importance of such intramolecular interactions in heteroleptic [Cu(P^P)(N^N)]^+^ complexes (see earlier discussion).^60^ This series of compounds displays strong blue-green or green emission (*λ*^em^_max_ = 489–539 nm) in the solid state, with PLQYs of 61% for [Cu(POP)(51)][PF_6_], 69% for [Cu(POP)(52)][PF_6_], 42% for [Cu(POP)(53)][PF_6_] and 58% for [Cu(POP)(54)][PF_6_]. The correlation of *λ*^em^_max_ with the electronic properties of the NHC ligands is not straightforward. However, DFT calculations indicate that both HOMO and LUMO contain NHC character giving a rationale as to why the observed trends in emission maxima do not follow directly from the electron-withdrawing/donating properties of the Me, OMe, F and Cl substituents. At 77 K, *λ*^em^_max_ values are red-shifted with respect to 298 K and emission decay times are longer. As for the parent compound [Cu(POP)(49)][PF_6_], it is concluded that emission at ambient temperatures has both phosphoresence and TADF components.^159^

More recently, this family of compounds has been extended by Zhao and coworkers to [Cu(POP)(55)][PF_6_] (*λ*^em^_max_(PL) = 493 nm at 298 K), and the photophysical properties were compared to those of [Cu(POP)(51)][PF_6_] (*λ*^em^_max_(PL) = 487 nm), again with the conclusion that both phosphorescence and TADF contribute to emission at ambient temperatures.^160^ We note that the solid-state emission behaviour of [Cu(POP)(51)][PF_6_] has been independently reported by Wang, Xu and coworkers^159^ and by Zhao and coworkers.^160^ In the initial report, the room temperature PLQY of solid [Cu(POP)(51)][PF_6_] was reported as 61%.^159^ In contrast, Zhao and coworkers determined a PLQY of 100%.^160^ Whether this is a consequence of different sample morphologies^32^ remains unclear. No applications of this family of four-coordinate [Cu(POP)(NHC)]^+^ emitters in LECs have been reported to date, despite the rather promising photophsyical behaviours.

## Conclusions

Over the last ten years, there has been growing interest in the development of luminescent heteroleptic copper(i) coordination compounds. These developments encompass both neutral compounds which may have applications in OLEDs and cationic complexes with potential use in either LECs or OLEDs. This review has focused mainly on Cu-iTMCs, but we have included neutral coordination compounds such as the series of [Cu(POP)(pz_2_BH_2_)], [Cu(POP)(pz_2_BPh_2_)] and [Cu(POP)(pz_4_B)]^62^ which were pivotal along the road to recognizing that heteroleptic Cu(i) complexes could exhibit TADF. Among the families of emissive Cu-iTMCs, the most commonly encountered are distorted tetrahedral [Cu(P^P)(N^N)]^+^ complexes in which P^P is a large-bite angle bis(phosphane) and N^N is a diimine or related ligand. Although initial investigations demonstrated high PLQYs and promising performances in LECs, it was not until low temperature PL measurements became routine that the widespread nature of TADF in Cu-iTMCs was recognized. Thus, in some cases, Cu-iTMCs were not described as TADF emitters even though the phenomenon was present.

There have been a number of endeavours to develop structure–property relationships to assist the synthetic chemist in ligand design,^57,60,102,154^ but often, simple ligands such as 6,6′-Me_2_bpy or 2,9-Me_2_phen have proved to be the best performing. It is critical that the N^N ligand possesses appropriate substituents to prevent flattening of the coordination sphere excited state. As far as wide-bite angle bis(phosphanes) are concerned, POP and xantphos remain the most popular, but RN-xantphos ligands (derived from the commercially available HN-xantphos) deserve further exploitation in [Cu(RN-xantphos)(N^N)]^+^ coordination compounds.

Most of the heteroleptic copper(i) complexes incorporating N- and P-donor ligands are red, orange or yellow emitters. Colour tuning into the blue region of the spectrum has been most notably achieved by moving to NHC-containing compounds. This area remains a valuable playground for the synthetic chemist.

Our longstanding interest in structural chemistry has led us to put an emphasis in this review on solid-state structural features such as π-stacking interactions and other inter-ligand interactions which may impact on PLQYs. Since it has been established that, for [Cu(P^P)(phen)]^+^ derivatives, there is a direct relationship between the number of intramolecular π-interactions in the ground state and the PLQY value,^60^ it is invaluable to correlate crystal-structural features with solid-state photophysical behaviour.

Finally, although we have, where possible, reported LEC performances, it is difficult to compare device figures-of-merit because of the differing LEC configurations and operating conditions. There are only a few investigations that focus on device optimization for a given Cu-iTMC electroluminophore, and we conclude that this is one area that is ripe for future development.

## Author contributions

C. E. H. and E. C. C. contributed equally to the writing of this review.

## Conflicts of interest

There are no conflicts to declare.

## Supplementary Material
